# Time-series satellite remote sensing reveals gradually increasing war damage in the Gaza Strip

**DOI:** 10.1093/nsr/nwae304

**Published:** 2024-08-24

**Authors:** Shimaa Holail, Tamer Saleh, Xiongwu Xiao, Jing Xiao, Gui-Song Xia, Zhenfeng Shao, Mi Wang, Jianya Gong, Deren Li

**Affiliations:** State Key Laboratory of Information Engineering in Surveying, Mapping and Remote Sensing, Wuhan University, Wuhan 430072, China; State Key Laboratory of Information Engineering in Surveying, Mapping and Remote Sensing, Wuhan University, Wuhan 430072, China; Geomatics Engineering Department, Faculty of Engineering at Shoubra, Benha University, Cairo 11629, Egypt; School of Computer Science, Wuhan University, Wuhan 430072, China; State Key Laboratory of Information Engineering in Surveying, Mapping and Remote Sensing, Wuhan University, Wuhan 430072, China; Collaborative Innovation Center of Geospatial Technology, Wuhan 430079, China; School of Computer Science, Wuhan University, Wuhan 430072, China; State Key Laboratory of Information Engineering in Surveying, Mapping and Remote Sensing, Wuhan University, Wuhan 430072, China; Collaborative Innovation Center of Geospatial Technology, Wuhan 430079, China; School of Computer Science, Wuhan University, Wuhan 430072, China; National Engineering Research Center for Multi-media Software, Institute of Artificial Intelligence, Wuhan University, Wuhan 430072, China; State Key Laboratory of Information Engineering in Surveying, Mapping and Remote Sensing, Wuhan University, Wuhan 430072, China; Collaborative Innovation Center of Geospatial Technology, Wuhan 430079, China; State Key Laboratory of Information Engineering in Surveying, Mapping and Remote Sensing, Wuhan University, Wuhan 430072, China; Collaborative Innovation Center of Geospatial Technology, Wuhan 430079, China; State Key Laboratory of Information Engineering in Surveying, Mapping and Remote Sensing, Wuhan University, Wuhan 430072, China; Collaborative Innovation Center of Geospatial Technology, Wuhan 430079, China; State Key Laboratory of Information Engineering in Surveying, Mapping and Remote Sensing, Wuhan University, Wuhan 430072, China; Collaborative Innovation Center of Geospatial Technology, Wuhan 430079, China

**Keywords:** war damage monitoring, satellite remote sensing, missile crater detection, type of damaged building, building damage level detection, Gaza Strip

## Abstract

War-related urban destruction is a significant global concern, impacting national security, social stability, people’s survival and economic development. The effects of urban geomorphology and complex geological contexts during conflicts, characterized by different levels of structural damage, are not yet fully understood globally. Here we report how integrating deep learning with data from the independently developed LuoJia3-01 satellite enables near real-time detection of explosions and assessment of different building damage levels in the Israel–Palestine conflict. We found that the damage continually increased from 17 October 2023 to 2 March 2024. We found 3747 missile craters with precision positions and sizes, and timing on vital infrastructure across five governorates in the Gaza Strip on 2 March 2024, providing accurate estimates of potential unexploded ordnance locations and assisting in demining and chemical decontamination. Our findings reveal a significant increase in damage to residential and educational structures, accounting for 58.4% of the total—15.4% destroyed, 18.7% severely damaged, 11.8% moderately damaged and 12.5% slightly damaged—which exacerbates the housing crisis and potential population displacement. Additionally, there is a 34.1% decline in the cultivated area of agricultural land, posing a risk to food security. The LuoJia3-01 satellite data are crucial for impartial conflict monitoring, and our innovative methodology offers a cost-effective, scalable approach to assess future conflicts in various global contexts. These first-time findings highlight the urgent need for an immediate ceasefire to prevent further damage and support the release of hostages and subsequent reconstruction efforts.

## Teaser

This study introduces a novel method combining deep learning and satellite data to precisely assess damage in conflict zones, revealing significant destruction in the Gaza Strip and highlighting the urgent need for intervention.

## INTRODUCTION

Violent armed conflicts, characterized by artillery shelling and bombings, exert grave and enduring humanitarian impacts on affected civilian populations. These impacts encompass loss of life and extensive damage to academic, scientific, economic and health infrastructures in conflict zones, thereby necessitating significant international attention [1–3]. The scarcity, contentiousness and often incomplete nature of data from conflict zones significantly hampers humanitarian relief efforts, the identification of potential international law violations, the enrichment of academic discourse on conflict resolution and the comprehension of societal impacts. The advent of open-source radar satellite data utilization marks a significant stride in the continuous monitoring of conflicts [4]. The general public’s access to these data and its bolstering of a burgeoning open-source community of researchers signify a crucial shift towards democratizing conflict analysis [5]. However, while the data offer a wealth of insights, their limited details impede the precise differentiation between military and civilian assets, a crucial factor for understanding conflict dynamics and humanitarian impacts. Detailed accuracy in data would markedly augment their utility in assessing infrastructure damage, pinpointing blast sites and evaluating environmental consequences. Without this level of detail, risks of misinterpretation loom, potentially leading to ineffective responses or misunderstandings. Hence, while satellite radar data are an invaluable resource, they should not be the sole reliance. For a comprehensive and objective analysis of conflicts, these data should be integrated with other information sources, such as high-resolution optical satellite imagery [6]. High-resolution satellite data have been utilized in numerous studies to assess and monitor damage in conflict areas [7–13]. Recent reports have combined high-resolution satellite data with artificial intelligence algorithms to identify destruction caused by natural disasters and to detect craters on the Moon and Mars [14–22]. For instance, these studies [23,24] combined remote sensing technology and artificial intelligence to analyze and assess the damage of natural disasters such as the 2023 Turkey–Syria earthquake, demonstrating approaches that can be adapted and expanded to address the unique challenges of conflict zones. Additionally, a recent study [25] explored the effects of conflict on malaria risk in sub-Saharan Africa and the broader impact of conflict on public health. However, the technical challenges associated with analyzing urban geomorphological structures and complex geological contexts during armed conflicts are characterized by various levels of structural damage and severe class imbalances among them, as well as the fact that impact craters resulting from heavy bombs, which differ significantly from those found on celestial bodies, are not yet fully understood globally. Here, we report on mapping war damage to critical infrastructure using a deep learning-based analysis technique from a time series of high-resolution LuoJia data [26,27] amid the recent escalation in the Israel–Palestine conflict. This report addresses six fundamental questions:

1. What is the impact of rocket shells on vital infrastructure, and how does the detection of missile crater positions contribute to understanding this impact?
2. Have the rates of building damage increased due to violent bombing during different periods?
3. How do the effects of rocket shells affect vital architectural styles such as hospitals, schools, mosques and cultural heritage, and their damage numbers by the war?
4. What are the proportions of different levels of damage to buildings in the war zones, and will they affect the housing crisis?
5. What is the proportion of agricultural land damage caused by the war, and does it affect food supplies?
6. What is the relationship between the spatial distribution of missile craters and the extent of damage to residential buildings and agricultural lands?

To map war damage, we created a dataset based on a time series of LuoJia3-01 [26] satellite data, with a spatial resolution of 0.65 m. We used all 40 images, each covering an area of $12\times 12\,{\rm km}^2$, acquired between 17 October and 2 March 2024 (Text S1). We developed an automated method to detect war damage to buildings and farmland and locate missile craters using these data. Our study area includes five governorates in the Gaza Strip, a region significantly affected by the Israel–Palestine conflict, which provides a crucial context for understanding the impact of the war on urban and community structures. In this study, we define urban annihilation by aerial or artillery bombardment as the systematic destruction of buildings, critical infrastructure and cultural heritage within conflict areas, which manifests as significant changes in the urban landscape detectable and measurable via high-resolution satellite imagery.

**Figure 1.**
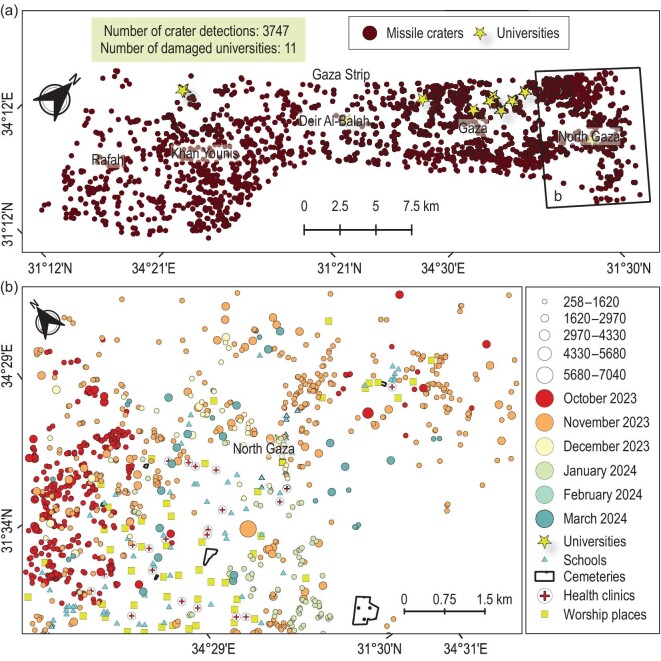
Automatic blasts map dated 2 March 2024 for the Israel–Palestine conflict. Panel (a) illustrates the detected missile craters in brown (circles). The locations of affected universities are indicated with yellow stars. Panel (b) highlights the northern Gaza area. Missile crater detections are coloured by date of occurrence and scaled by diameter (centimetres). Locations of health clinics, schools, worships and cemeteries subjected to damage are also clarified.

However, discerning whether an aerial missile or ballistic bomb is causing civilian casualties or significant environmental and chemical pollution cannot be ascertained from satellite data alone. We identified areas affected by rocket-propelled grenades (i.e. areas where bomb craters have been detected) and determined the positions, numbers, sizes and timings of missile craters at the conflict level. We found 3747 missile craters across five governorates in the Gaza Strip, which serve as a historical record in determining accurate estimates of potential unexploded ordnance (UXO) locations and assisting in demining and chemical decontamination. Among these five governorates, the North Gaza Governorate was notably severely affected by missile craters, indicating significant armed violence (Fig. 1). We also manually estimated the missile craters, using this as a reference for verification. Validation of independent satellite samples tested via several visual inspection techniques demonstrated an overall accuracy level of over 95% for our method (Table S2).

**Figure 2.**
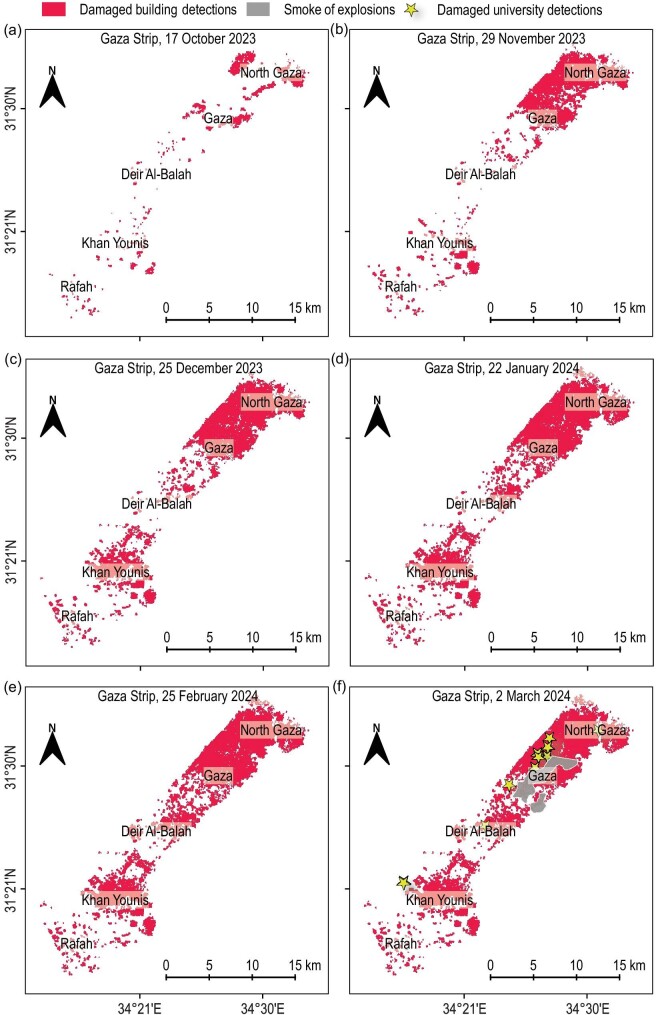
A map for automatically detecting damage across different periods. The areas of detected building damage are indicated in red, while universities are marked with yellow stars. Panel (a) illustrates the onset of conflict on 17 October 2023, where the damage rate was 14% compared to the pre-conflict reference image dated 7 May 2022. Panel (b) captures a period of escalated hostilities in the north, after the announcement of a prisoner exchange truce by both sides on 24 November 2023, evidenced by a damage rate of 32.6% on 29 November 2023. Panel (c) shows the aftermath of intensive military operations in the south on 25 December 2023, with a damage rate of 47.9%. Panel (d) represents the effects of pervasive bombing across the entire Strip on 22 January 2024, culminating in a total building damage rate of 56.0%. Panels (e) and (f) visualize the intensive operations targeting buildings in the central Strip on 25 February and 2 March 2024, where the damage rate reached 58.2% and 58.4%, respectively. Gray-shaded areas in panel (f) denote regions obscured by dense blast smoke.

**Figure 3.**
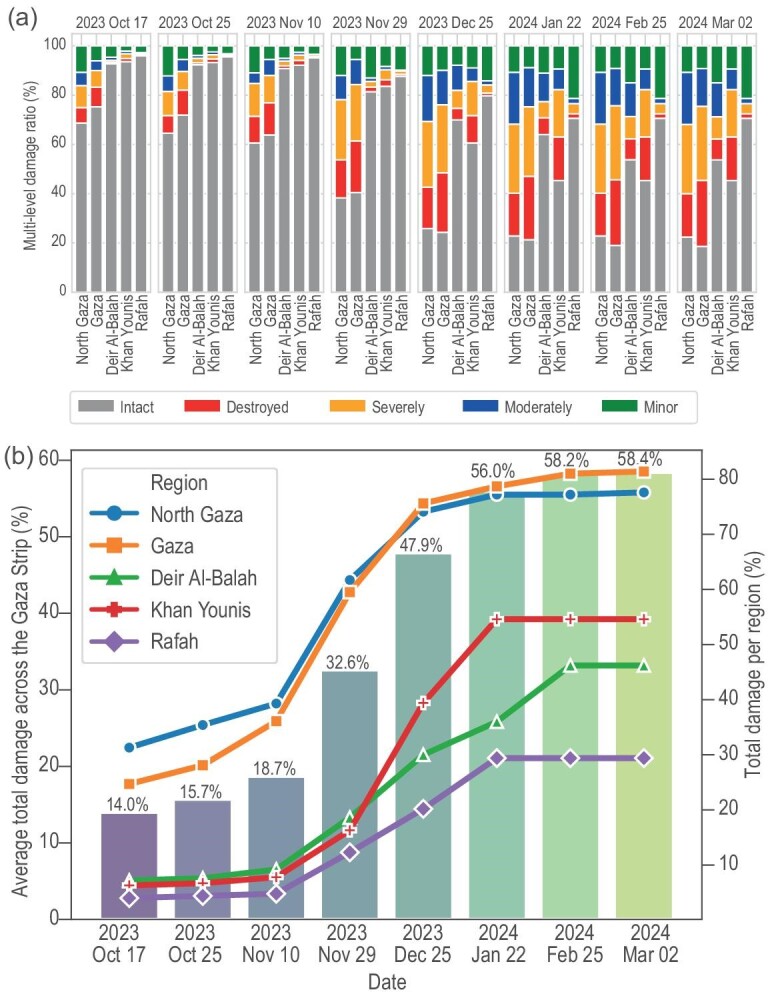
A detailed time-series analysis illustrating the extent of building damage in the Gaza Strip from 17 October 2023 to 2 March 2024. Panel (a) provides a granular breakdown of multi-level damage rates across five governorates, highlighting a significant reduction in the number of intact housing units, which underscores the severe impact of the conflict on residential infrastructure. Panel (b) depicts a comparative examination between the average percentage of total damage across the Gaza Strip and damage incurred in each governorate over time. Notably, the North Gaza and Gaza governorates have experienced the most substantial increase in damage rates to housing units. This observation is indicative of the intense and sustained hostilities within these areas during the analyzed period, reflecting the dire humanitarian and housing crises emerging from the conflict.

On the other hand, our approach facilitates the monitoring of different levels of building damage (destroyed, severe, moderate, slight) by integrating data on building damage resulting from natural disasters such as earthquakes and global floods [21,22] within a Siamese network (Text S4). This methodology was used to examine the spatio-temporal dynamics of conflict intensity over the urban environment. From 17 October 2023 to 2 March 2024, we found that the damage continually increased to residential and educational buildings, which totalled 213.16 km$^2$, equivalent to about 58.4% of the Gaza Strip’s total area of 365 km$^2$ (Fig. 2). This situation has resulted in a crisis characterized by a lack of safe housing and an unprecedented displacement of residents due to violent artillery and missile shelling, providing insights into post-war reconstruction efforts and urban and rural planning. We found that 15.4% of structures were destroyed, 18.7% severely damaged, 11.8% moderately damaged and 12.5% slightly damaged. The North Gaza Governorate, accounting for 77.6% of the total damaged area (47.5 km$^2$), and the Gaza Governorate, with 81.4% of the total damaged area (60.1 km$^2$), experienced the most extensive destruction. By contrast, the Deir al-Balah and Khan Yunis governorates saw an average of more than 50% of buildings damaged. The Rafah Governorate witnessed less damage, accounting for 29.4% of the total area of damaged buildings (Fig. 3).

Additionally, we identified 101 hospitals and medical clinics, 283 places of worship, including mosques and churches, 295 educational institutions, encompassing kindergartens, schools and universities, and 32 cemeteries, all of which sustained major or minor damage (Fig. S6). This destruction has complicated the community’s ability to access medical treatment and medication, practice spiritual life and access education, and conduct burial ceremonies. These challenges are a direct result of the brutal actions taken by the Israeli occupation army against the Palestinian people. Furthermore, there was a decrease of 62.9 km$^2$, or 34.1%, in the cultivated area of agricultural land, from a total of 184.7 km$^2$, due to bulldozing and rocket bombs. This reduction in the cultivated area during the conflict in February 2024 has led to a significant decline in local agricultural output, causing serious humanitarian issues for food security in the conflict zones. We have concluded that the rapid increase in damage in the main areas of the Gaza Strip is attributable to five principal types of urban destruction, including aerial bombardment, artillery shelling, bulldozing, mine blasting and infrastructure sabotage. These actions are driven by factors such as occupying lands due to their clear strategic importance, ethnic tensions or retaliatory measures amid intense military clashes. We also identified a relationship between the incidence of destruction in densely populated and rural areas within the conflict (specifically within agricultural land), which leads to higher rates of population displacement compared to more isolated or rural areas ($P< 0.001$; Fig. S9). This finding underscores the crucial role of strategic importance and population density in targeting and displacement patterns during conflicts. It is expected that the intensity of the war will escalate in conflict areas, causing significant environmental and humanitarian damage. Furthermore, the LuoJia3-01 satellite data are crucial for impartial conflict monitoring, and our innovative methodology offers a cost-effective, scalable approach to assessing future conflicts in various global contexts. These first-time findings highlight the urgent need for an immediate ceasefire to prevent further damage and support the release of hostages and subsequent reconstruction efforts. The remainder of the paper is structured as follows: the results section discusses crater detection, building damage detection and change detection in agricultural land. This is followed by the discussion and conclusion section, and finally, the methods section.

## RESULTS

### Crater detection

The LuoJia3-01 satellite imagery confers a unique advantage in accurately identifying bomb craters resulting from aerial campaigns, distinguishing it from the publicly accessible Sentinel-1 and Sentinel-2 satellites. Our investigation identified 3747 craters attributable to missile strikes between 17 October 2023 and 2 March 2024, spanning approximately 141 square miles across various governorates in the Palestinian territories, including North Gaza, Gaza City, Deir Al-Balah, Khan Younis and Rafah (Fig. 1). A demonstration video showcasing the detected craters is available in the online supplementary material (Movie S1). These detonations impacted diverse settings, including densely populated urban locales, roads, bridges and agricultural domains.

Moreover, our analysis discerned variations in blast magnitudes, with elaborative details presented in Table S2. Before the conflict, data processing until 7 May 2022 uncovered an additional 17 craters, potentially indicative of collapsed tunnels or infrastructure foundations within the Gaza Strip (Fig. S4). After the conflict, analysis unveiled pronounced military engagement, notably within the North Gaza Governorate, where a heightened scouting capacity was observed, signaling intense combat persisting until early March 2024. A detailed temporal examination illustrates fluctuations in the detected blast counts, correlating with specific incidents. Between 17 October 2023 and 2 March 2024, we detected an average of 1036 blasts in the North Gaza Governorate, with the highest activity recorded on 10 November, when 357 blasts were identified. Subsequently, the number of blasts detected on 29 November 2023 decreased to 40%, coinciding with the announcement of a prisoner exchange truce [28] between Israel and Hamas on 24 November 2023. Nonetheless, on 29 November 2023 attacks were detected that resulted in 143 blasts. By 25 February 2024, the number of detected blasts had diminished to only four. On 2 March the number of detected blasts rose again to 36. This coincided with the violence on 29 February 2024, when thousands rushed toward food aid [29]. The missile strikes also targeted critical infrastructure throughout the Gaza Strip, affecting the seaport and various educational, health and religious facilities (Fig. S3).

Following the announcement by the Israeli army of its withdrawal from northern Gaza near the end of December 2023 [30], our analysis expanded to include additional governorates, revealing ongoing aggressive actions. Notably, between 25 December 2023 and 22 January 2024, we detected 872 blasts in the Khan Younis Governorate in the south (Table S3). Additionally, we assess the efficacy of manual detection versus the automated detection method (Fig. S4). The trained model demonstrated the capability to accurately identify craters, achieving an F1 score of 84.3%, which underscores the artificial intelligence model’s proficiency in crater detection, although its performance is contingent upon the crater’s size. For instance, for craters with diameters less than 5 m, the F1 score is 76% per crater. The analysis further explores the challenge of deducing the type of munition from imagery and associating the blast’s size with the characteristics of the potential munition to ascertain the extent of the damage.

An analysis posted by *The Washington Post* [31] suggests that any blast exceeding 12.2 m (40 feet) in diameter likely originates from a bomb weighing at least 2000 pounds. Such a munition poses a significant threat to civilians and can inflict extensive and irreversible damage to structures, potentially leading to devastation if deployed from an altitude of 90 m or higher. This raises the question: what implications arise if the blast’s diameter surpasses 70 m, as depicted in Fig. S4, point E and Fig. S5e? These observations highlight the importance of detailed mappings in facilitating mine clearance operations, UXO risk assessments, chemical decontamination and post-conflict reconstruction initiatives in Palestine, thereby illustrating the pivotal role of advanced satellite imagery in the analysis of contemporary conflicts and humanitarian endeavours.

### Building damage detection

**Figure 4.**
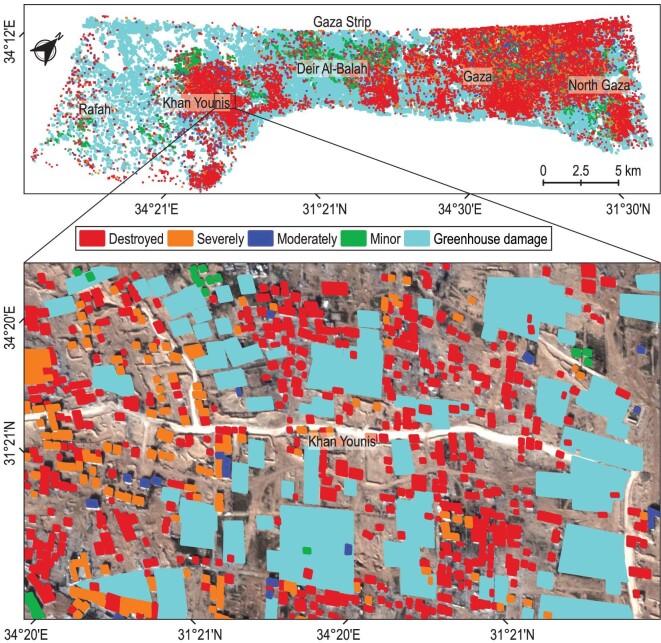
Automatic detection map for different levels of damage for 2 March 2024. This figure illustrates the detected damages, categorized by type and severity. Buildings are classified according to their damage level: destroyed (red), severely damaged (orange), moderately damaged (blue), minor damage (green) and greenhouse damage (cyan).

Although many computer vision-based methods have demonstrated promising accuracy in detecting building damage resulting from natural disasters such as earthquakes and floods [14–17], estimating demolished building damaged by intense armed violence using satellite data remains a challenging research area. The significant class imbalance in urban conflict zones, coupled with the considerable diversity in building shapes, sizes and varying lighting conditions, complicates the classification process. In this study, we focus on detecting building damage associated with armed conflict by applying automated deep learning techniques, complemented by our estimation of multiple levels of damage categories for each building. To enhance detection accuracy, we have integrated multi-scale features and cross-directional attention mechanisms into our methodology (Text S4). Our estimates indicate that, as of 2 March 2024, approximately 58.4% of the total housing units in the Gaza Strip were completely or partially destroyed since the conflict’s onset (Fig. 2). We identified approximately 168 170 damaged buildings out of a total of 288 000 buildings in the Gaza Strip, rendering them uninhabitable. This total includes 44 305 destroyed buildings, 53 975 severely damaged buildings, 34 020 moderately damaged buildings and 35 870 slightly damaged buildings. These figures underscore a significant and urgent humanitarian concern in the region. Notably, the North Gaza and Gaza governorates have experienced a substantial increase in damage. In the North Gaza Governorate, we identified at least 35 700 damaged buildings, constituting 77.6% of the total buildings (46 000) in the governorate as of 2 March 2024, including 8100 destroyed buildings, 12 960 severely damaged buildings, 9740 moderately damaged buildings and 4900 slightly damaged buildings (Fig. S4). In the Gaza Governorate, we identified a total of 50 450 damaged buildings, accounting for 81.4% of the governorate’s total area, including 16 590 destroyed buildings, 18 700 severely damaged buildings, 9480 moderately damaged buildings and 5680 slightly damaged buildings. Among these, educational facilities, health facilities, houses of worship, government buildings and charitable institutions suffered extensive damage. We found 101 hospitals and medical clinics that suffered major to minor damage. Spiritual life centres were not spared; we documented damage to 283 houses of worship, including mosques and churches. Educational institutions, numbering 295, including schools and universities, also sustained damage. Additionally, the conflict affected the resting places of the dead, as there were 32 damaged cemeteries, complicating the community’s ability to conduct burials (Fig. S6). These statistics highlight the profound and extensive consequences of conflict on both the physical infrastructure and the social fabric of affected communities. Moreover, time-series analysis reveals a clear pattern of escalating building damage rates across the five governorates, with the North Gaza region exhibiting the most significant increase during the observed period. This increase is particularly pronounced, rising from 31.3% on 17 October 2023 to 77.6% on 2 March 2024 (Figs 3 and 4), indicating a significant rise in the number of destroyed housing units in this governorate. The Gaza Governorate has seen a steady increase in damage rates, rising from 24.7% on 17 October 2023 to 81.4% on 2 March 2024. Khan Yunis has also experienced an increase in damage rates, from 6.3% on 17 October 2023 to 54.6% on 2 March 2024. The overall damage percentage has risen over the observed period, from 14.0% on 17 October to 58.4% on 2 March. These findings underscore the escalating impact of violent conflict on critical infrastructure from 17 October 2023 to 2 March 2024, with thousands of housing units and essential facilities damaged, leading to an acceleration in the number of civilian casualties. Additionally, the number of refugees displaced by the conflict has increased, forcing people to seek refuge in safer areas, exacerbated by the absence of truly safe places within Gaza (Fig. S8). Consequently, our method proves to be an effective tool for the objective, real-time monitoring of the ongoing conflict, offering valuable insights to advocate for an immediate humanitarian ceasefire, guide critical infrastructure reconstruction efforts and garner support from the international community and governments to achieve peace and revive the Strip.

### Changes in agricultural land

**Figure 5.**
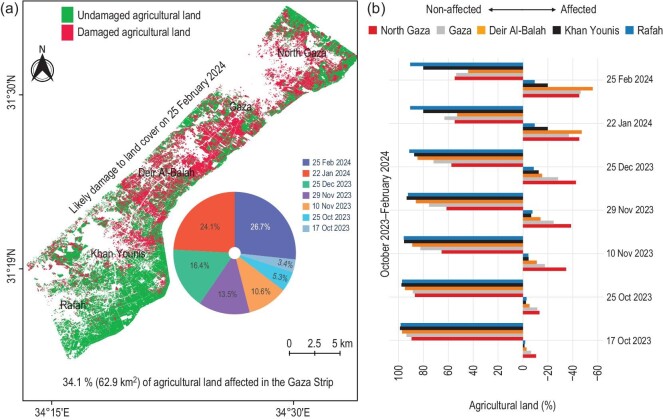
Time series of affected agricultural land during the Israel–Palestine conflict spanning from 17 October 2023 to 25 February 2024. (a) Comparison of cropland change with the previous year (2022), indicating that the overall affected cropland constitutes 34.1% (62.9 km$^2$) of the total 184.7 km$^2$. (b) Percentage reduction of agricultural land on various dates during the conflict, delineated for each governorate in the Gaza Strip.

We employed a multi-temporal random forest classification approach to characterize changes in agricultural areas over specific time frames. Figure 5 illustrates notable changes in the Gaza Strip’s agricultural areas due to land leveling during the ongoing conflict. Before the conflict, the cultivated area in the Gaza Strip was 184.7 km$^2$, constituting approximately 50.6% of the total area of the Strip. In contrast, during the conflict, the area of agricultural land decreased by 34.1%, which means that 62.9 km$^2$ of the total cultivated area was affected by 25 February 2024. Our assessment included an estimate of the average damage to agricultural land in the governorates of North Gaza, Gaza City, Deir Al-Balah, Khan Younis and Rafah, which amounted to more than 45.2%, 46.4%, 56.0%, 19.9% and 9.4%, respectively.

These results underscore that the change in the area of agricultural land affected during the ongoing conflict is more pronounced than in the period before 7 October 2023, resulting in a significant loss of agricultural land. This loss is exacerbated by various factors, including soil compaction by heavy vehicles and tanks, necessitating the use of heavy tillage for future agriculture—a significant challenge given the lack of such machinery in the Gaza Strip. Moreover, the destruction of vegetation and tree cover increases the land’s vulnerability to desertification, which, in turn, accelerates soil erosion during rainfall. The destruction of fruit seedlings, olive trees and crops further compounds the issue, as the surface soil cover is disturbed, affecting future agricultural prospects. The cumulative impact of these deteriorations leads to high repair costs and a long-term decline in agricultural productivity, contributing to the exacerbation of the ongoing humanitarian crisis and underscoring the urgency to rebuild and halt the conflict.

## DISCUSSION AND CONCLUSION

Our analysis of the Luojia3-01 time-series data, spanning from 17 October 2023 to 2 March 2024, during the ongoing Israel–Palestine conflict, revealed the dataset’s inaugural application for real-time monitoring of conflict dynamics. This novel utilization of LuoJia3-01 data facilitates the creation of detailed maps illustrating the extent of the destruction. These maps depict demolished or damaged buildings, the distribution of identified missile craters and their impacts on agricultural lands, closely correlating with areas experiencing intense hostilities, missile strikes, bulldozing operations and artillery assaults. The quantitative study of this form of intense armed violence, beyond specific case studies, is currently impossible due to the absence of systematic data.

In this paper, we combine artificial intelligence techniques and high-resolution satellite imagery to automatically detect signs of destruction resulting from Israeli aggression on the Palestinian Gaza Strip, such as collapsed buildings, missile craters and affected agricultural land. This approach provides practical benefits to both practitioners and researchers. While these results are promising, real-world monitoring requires high precision, especially when destruction is scattered across the conflict zone and detecting destroyed buildings is akin to searching for a needle in a haystack. We demonstrate that exploiting the time-series details of continuous satellite images of building destruction data can significantly improve the training algorithm for automated destruction monitoring and even approach real-time tracking for policy purposes.

We make four relevant methodological contributions to the field of conflict damage assessment and destruction detection. First, we leveraged LuoJia3-01 satellite data to develop a comprehensive and objective dataset of high-resolution satellite imagery within an active conflict zone, enabling near real-time detection of destroyed infrastructure, affected farmland and monitoring of crater sites caused by explosive bombs (Text S2). Second, we presented a novel Siamese method to detect changes in buildings damaged by the Palestinian–Israeli conflict, mapping entire populated areas in the Gaza Strip, and assessing damage quickly and accurately, overcoming the limitations of traditional manual inspection methods, which are time consuming and dangerous. Third, we integrated an attention mechanism based on the temporal dimension of images to highlight subtle differences in damage levels, coupled with data augmentation strategies to mitigate class imbalance issues. Fourth, we developed an extensive database of missile craters, precisely annotated to include a wide range of shapes, sizes and morphological features. This database significantly enhances the robustness and generalizability of our detection model in automatically detecting missile crater locations and sizes (Texts S3 and S4).

Thanks to these advances, our automated methodology for heavy missile crater identification yields precise spatial and attribute data for each detected crater, boasting an impressive F1 score of 84.3% for five governorates in the Gaza Strip, indicative of high accuracy and reliability. Moreover, we demonstrate that our methodology is a cost-effective and rapid method to detect and quantify varying damage levels to buildings, enabling a thorough assessment of destruction levels. This analytical framework is versatile, applicable globally across different regions, or with alternative high-resolution sensor data, to monitor and evaluate destruction in various conflict contexts. Furthermore, the data’s accuracy offers unique insight into the types of artillery or ammunition used, aiding in the identification of potential international law violations.

While our blast detection (crater detection) database may not be exhaustive, it exemplifies a level of comprehensiveness and objectivity surpassing the scope of publicly reported attacks, thereby underscoring its value in augmenting external reports and establishing itself as a dependable data repository for initiatives such as UXO and chemical decontamination efforts. By taking advantage of the number of missile craters we have detected (a proxy for exploding bombs) in the target area, the number of exploding bombs can be subtracted from the total bombs dropped, thus estimating the number of bombs that remain unaccounted for and are likely hidden in the conflict area. To date, we have no information on publicly available reports detailing the exact total number of bombs dropped, which opens horizons for future work in identifying UXO estimation. A potential future improvement of our method could be to incorporate data from other remote sensing satellites, such as synthetic aperture radar and the normalized difference built-up index, to further enrich the contextual information of land surfaces and provide a more comprehensive understanding of damage in urban and agricultural settings. Finally, this facet of the analysis not only underscores the significance of LuoJia3-01 time-series data in conflict monitoring, but also highlights its potential to support adherence to international humanitarian standards.

## METHODS

Detailed descriptions of all methods and materials are presented in the online supplementary material. Briefly, the LuoJia3-01 satellite [26] is used to track active conflict areas and monitor damages (Text S1). The raw satellite data are pre-processed by dividing them into smaller patches and creating masks with different levels of damage (Text S2). Given the high cost of annotating the destruction effects, we focused on the North Gaza Governorate, which experienced severe violence and significant destruction, and created corresponding ground annotations for training. Figure S13 shows the full extent of the North Gaza Governorate, with annotations depicting different levels of destruction. The differences in the model’s predictions for destroyed areas before and after manual examination are shown in panels (b) and (c) of Fig. S13. The statistical measures for the damaged buildings are provided in Table S4.

Additionally, a validation test using the receiver operating characteristic curve is conducted in Fig. S14, demonstrating the advantages of ground truth in model evaluation for conflict areas not included in the training sample. The missile craters are automatically detected from satellite imagery using a single-stage deep learning-based detector. The detected craters are then exported in GeoJSON format, allowing for seamless integration with the QGIS platform and enabling in-depth spatial analysis (Text S3). Moreover, building damage detection is performed by developing a Siamese convolutional neural network with semi-supervised learning (Text S4). Agricultural land masks are created by calculating the normalized red-green difference index before and after the conflict, enabling the detection of changes in farmland areas for annotation purposes. Finally, these annotations are used to train a random forest classification algorithm to distinguish between affected and unaffected farmland, assessing their potential impact on agricultural productivity (Text S5).

## Supplementary Material

nwae304_Supplemental_File

## Data Availability

All data originally used in this study are accessible to the public upon reasonable request. The authors have made a GitHub repository public at https://github.com/oshholail/War-Damage to generate results according to the methods described in this manuscript. Note however that this repository is under development, so the network architecture is likely to change.
