# Supplementary material for: Time-series satellite remote sensing reveals gradually increasing war damage in the Gaza Strip
Source: Natl Sci Rev. 2024 Aug 24;11(9):nwae304. doi: 10.1093/nsr/nwae304 (PMC11413530; doi:10.1093/nsr/nwae304)
Supplement: nwae304_Supplemental_File [file nwae304_supplemental_file.pdf]

NSR National Science Review

Supporting Information for

Time-series Satellite Remote Sensing Reveals Gradually Increasing War Damage in the Gaza Strip

Shimaa Holail[1,†], Tamer Saleh[1,2,4,†], Xiongwu Xiao[1,3,†,*], Jing Xiao[4,†], Gui-Song Xia[1,3,4,5], Zhenfeng Shao[1,3], Mi Wang[1,3], Jianya Gong[1,3], Deren Li[1,3,*]

[1]State Key Laboratory of Information Engineering in Surveying, Mapping and Remote Sensing, Wuhan University, Wuhan 430072, China

[2]Geomatics Engineering Department, Faculty of Engineering at Shoubra, Benha University, Cairo 11629, Egypt

[3]Collaborative Innovation Center of Geospatial Technology, Wuhan 430079, China

[4]School of Computer Science, Wuhan University, Wuhan 430072, China

[5]National Engineering Research Center for Multi-media Software, Institute of Artificial Intelligence, Wuhan University, Wuhan 430072, China

† Equally contributed to this work.

Corresponding authors Email: xwxiao@whu.edu.cn; drli@whu.edu.cn

Contents of this file

Text S1 to S5
Figures S1 to S14
Tables S1 to S5
Legend for Movie S1
SI References

Other supporting materials for this manuscript include the following:

Movie S1

Text S1. Materials

High-resolution satellite data were utilized in this study to monitor the damage resulting from the Israeli-Palestinian conflict. The data were collected from the Chinese LuoJia3-01 (1) satellite of Wuhan University, which are designed to monitor and track active conflict zones globally in real-time. The specifics of these satellites are detailed in Table S1. Initially, pre-conflict imagery was sourced from Google Earth, an openly accessible platform, on May 7, 2022, with a spatial resolution of 1 m. Conflict period data, or damage images, were directly obtained from the Chinese satellite LuoJia3-01, covering the duration from October 17, 2023, to March 2, 2024, with an exceptional resolution of 0.65 meters. These original images encompass 40 snapshots of the Gaza Strip envelope, captured over several days. Each image is approximately 480 MB in size and contains about 315 million pixels with RGB bands. Some images show overlap, while others have fully or partially black/white pixels in the conflict zones, likely due to bomb smoke and explosions. This diverse time series of images aids in creating damage maps, assessing the condition of buildings and farmland, and identifying missile blast locations.

All data underwent resampling processing within the Universal Transverse Mercator (UTM) Zone 36 N using the open-source QGIS platform (https://www.qgis.org/en/site/). Data on administrative boundaries were sourced from the GADM database version 2.8 for global administrative regions (https://gadm.org/download_country_v2.html), noting that land cover data for the study area were excluded. Additional data, such as vector files for land cover, were acquired from the Open Street Map (OSM) service (https://data.nextgis.com/en/region/custom/base). Our analysis focused on evaluating the conflict's impact on infrastructure over six consecutive months: October, November, and December 2023, and January, February, and March 2024. This assessment involved calculating the total affected areas and the damage percentage in each governorate within the Gaza Strip during these specific time frames.

Text S2. Data Annotations

Generally, data about land cover and buildings, which are accessible from public sources such as Open Street Map (OSM), can be utilized as annotations for buildings. However, the utility of this data is constrained due to significant registration discrepancies between the buildings and satellite imagery, leading to considerable inaccuracies in the model. Regrettably, the raw post-conflict data from the LuoJia3-01 satellite lacks the essential ground truth information required for model training. A practical solution involves training the proposed model on an internationally recognized public dataset on natural disasters, the XBD dataset (available at https://xview2.org/dataset), which includes various types of natural disasters and provides a sample of 98,000 buildings annotated with different levels of damage. The model performed well on the XBD dataset; however, its results were not satisfactory in predicting damage types in an active conflict environment like the Israeli-Palestinian war. This outcome was expected, given the complexity of automated monitoring over time due to the lack of training data and the limited number of damage labels available for conflict areas, leading to potential machine learning overfitting. Notably, the XBD dataset did not include data on wars or complex urban environments like Gaza.

Additionally, the Gaza dataset is complicated by the proximity of buildings, making it challenging to distinguish between individual building's joints and alleys, along with colour variations and similarities. To address this issue, we enhanced our network's capabilities through fine-tuning by combining the XBD disaster dataset with destruction data from the Gaza war to improve the training process. We initially processed the raw data by dividing it into smaller patches using an image cropping tool. The optimal size of the cropping window is determined by factors such as the size of the buildings and the resolution of the image. The original set of 18,000 images was reduced to 1024 $\times$ 1024 pixels. These cropped images were then subjected to a filtering process to enhance dataset quality, which involved excluding images with significant smoke from explosions. After this data cleansing process, our dataset was composed of 7100 images, each associated with unique geographic coordinates. This curated dataset was employed to train a model using the XBD data, producing mask outputs that identify damaged buildings at the pixel level and categorize the extent of the damage. Given the high cost of annotating the destruction effects, we focused on the North Gaza Governorate, which experienced severe violence and significant destruction and created corresponding ground annotations for training.

Damage labels were created as six-channel masks, where channel 0 denotes the background, channel 1 represents destroyed buildings, channel 2 indicates buildings with major damage, channel 3 signifies buildings with moderate damage, channel 4 denotes buildings with minor damage, and channel 5 identifies intact buildings. The complete source code is available on GitHub (https://github.com/DIUx-xView/xView2_baseline) for generating mask outputs and damage predictions. These outputs were initially treated as ground truth for the positive label (building destroyed/severely/partially/slightly damaged).

To refine accuracy, a manual examination of these outputs was conducted through visual interpretation to ensure a more precise damage assessment, supplemented by building destruction annotations provided by UNOSAT (2). Concurrently, buildings extracted from pre-conflict images were used to create negative data (labelled as intact buildings). These extracted structures were then superimposed onto post-conflict images to assess and categorize building damage, adjusting their classification as needed to accurately reflect the observed damage. The full extent of North Gaza Governorate, with annotations depicting different levels of destruction, and differences between models for damaged areas before and after the manual examination are shown in Fig. S13. The statistical measures of damaged buildings are presented in Table S4. We also performed a validation test using the receiver operating characteristic (ROC) curve in Fig. S14, to demonstrate the advantages of ground truth in model evaluation in conflict areas not included in the training sample.

Text S3. Craters detection model simulation

Within the domain of contemporary conflict analysis, notably in the active Israeli-Palestinian conflict zone, an unreported number of detonated bombs have created craters, alongside a potentially substantial quantity of unexploded ordnance, presenting significant challenges to the environment, infrastructure, and post-conflict demining efforts. The conventional manual approach to crater identification and measurement is labour-intensive and prone to subjective biases. In contrast, automated crater detection methodologies employing artificial intelligence offer a systematic and objective alternative for the identification, mapping, and analytical assessment of craters caused by missile and artillery bombardment. Utilizing high spatial resolution satellite imagery, such as from LuoJia3-01 satellite, we have developed a new deep learning tool tailored to meet the unique demands of analyzing active conflict zones. The potential applications of this tool are manifold, ranging from aiding in the critical task of detecting and removing potential unexploded ordnance to providing detailed insights into the geospatial dynamics of conflict, including soil property assessment and subsurface structure analysis. Our methodology is inspired by the techniques outlined in (3, 4), which apply deep learning to autonomously recognize and pinpoint objects of interest. Initially, we utilized the red, green, and blue bands of the visible spectrum as our foundational band combination. Subsequent image correction was conducted using a rational polynomial coefficients (RPC) file, commonly known as a "rpb" file, to orthorectify the satellite images. This orthorectification is crucial for transforming image coordinates into precise ground coordinates. In training our detection algorithm, we employed a diverse array of missile crater images, precisely annotated to encompass a broad spectrum of shapes, sizes, and morphological features (Fig. S10). This variety is essential for developing a detection model that is both robust and generalizable across various contexts.

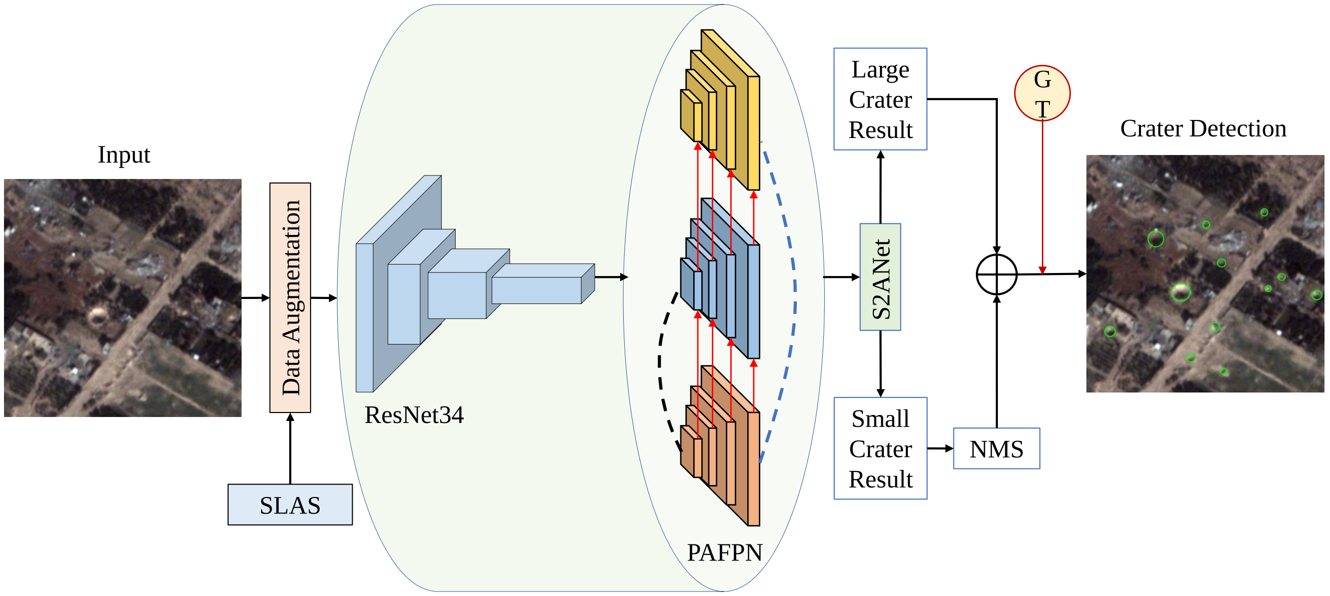

Fig. S1. The overall framework of the proposed crater detection approach.

The data was divided into training, validation, and testing sets to facilitate exhaustive experiments and identify the optimal model. For the model input, we utilized images resized to 1024×1024 pixels with an average spatial resolution of 0.65 m. Fig. S1 shows the overall architecture of the proposed approach to missile crater detection. We

first merge the LuoJia3-01 training dataset after pre-processing. To prevent model over-fitting, data augmentation (5) was applied to the combined training dataset, including random adjustments to the input images, such as horizontal and vertical shifts, rotations, and linear contrast enhancements. Also, the size of the minority class is increased by applying Self-Learning Augmented Segmentation (SLAS) (3). The main difficulty in detecting missile craters in high-resolution remote sensing images is the relatively small size and complex environment of the craters in the images. In this approach, a single-stage detector, S2A-Net (6), is trained on the training set. We chose S2A-Net with compatible convolutional features because it has excellent advantages in producing proposals for small-scale object classes such as cars and fishing boats. To extract features from remote sensing images, the ResNet34 (7) architecture is adopted as the backbone to create the underlying convolutional layers of the Feature Pyramid Network (FPN) (8).

We chose ResNet34 (7) because it is fast and flexible in identifying complex patterns in training data and extrapolating results effectively, while also having a low computational cost. The FPN enhances the standard convolutional network with a top-down pathway and lateral connections so that the network efficiently constructs a rich, multi-scale feature pyramid from a single-resolution input image. Although the traditional FPN excels at integrating higher-level semantic information with the multi-layer feature extraction network, it tends to lose low-level location information. Therefore, we further enhance the features through the path aggregation feature pyramid network (PAFPN) (9), which preserves the precise location information in the underlying features and shortens the information path between the lower layers and the upper features.

After obtaining the detection results, we use non-maximum suppression (NMS) (10) to improve the detection ability of the small missile crater array. Finally, we convert the coordinates of the detected bounding boxes from the rectangular shape (x_1, y_1, x_2, y_2) to the circular shape (x, y, r) using the geometric transformation (GT) as in Eq. 1 and obtain the crater detection results.

$$\begin{aligned} x &= \frac{x_1 + x_2}{2}, \\ y &= \frac{y_1 + y_2}{2}, \\ r &= \frac{(x_1 - x_2) + (y_1 - y_2)}{4}. \end{aligned} \qquad [1]$$

Here, x_1 and y_1 are the coordinates of the top left corner, x_2 and y_2 are the coordinates of the bottom right corner, x and y denote the coordinates of the circle center, and r is the length of the corresponding radius.

We also conducted some comparisons, including training and evaluating crater detection models from satellite images, using backbones such as ResNet34 (7), VGG16 (11), and InceptionV3 (12). Experiments were performed for 30 epochs, and validation results were recorded for each model. The main performance metrics used for evaluation include precision, recall, and F1-score as shown in Table S5. ResNet34 performs better than the other two models. It received the highest F1 scores in both its two conflict images dated November 29, 2023, and January 1, 2024, indicating balanced accuracy and recall. This distinction highlights how flexible ResNet34 is in identifying complex patterns in training data and effectively extrapolating results. As a result, ResNet34 is the preferred model for our specific task because it strikes a balance between high precision, recall, and accuracy and provides insight into deployment decisions that are useful in real-world applications. Employing the finely tuned model, we successfully automated crater detection within the conflict zone and exported the findings in GeoJSON format for seamless integration with the QGIS platform, enabling in-depth spatial analysis. Each detected crater was precisely geolocated, timed, and measured to compile a comprehensive dataset, facilitating inferences about the types of munitions used and the potential locations of unexploded ordnance, thereby assessing their impact on civilian safety throughout the conflict zone. To ensure the utmost accuracy, all detected craters were precisely reviewed to eliminate false positives, which could be mistaken for missile craters due to similarities to shadows, water towers, or remnants from past conflicts. Our methodology signifies a substantial advancement in conflict zone analysis, offering a precise, scalable, and unbiased tool for crater detection and analysis in active conflict zones. This innovative approach not only augments our understanding of conflict's immediate impacts but also contributes to a more profound comprehension of its long-term environmental and infrastructural repercussions, laying a solid groundwork for future research and intervention strategies in analogous global contexts.

Text S4. Building damage detection model simulation

Fig. S2 presents a comprehensive overview of the proposed framework designed for the assessment of battle damage on residential buildings. The framework is comprised of a pre-trained neural network tasked with predicting the state of

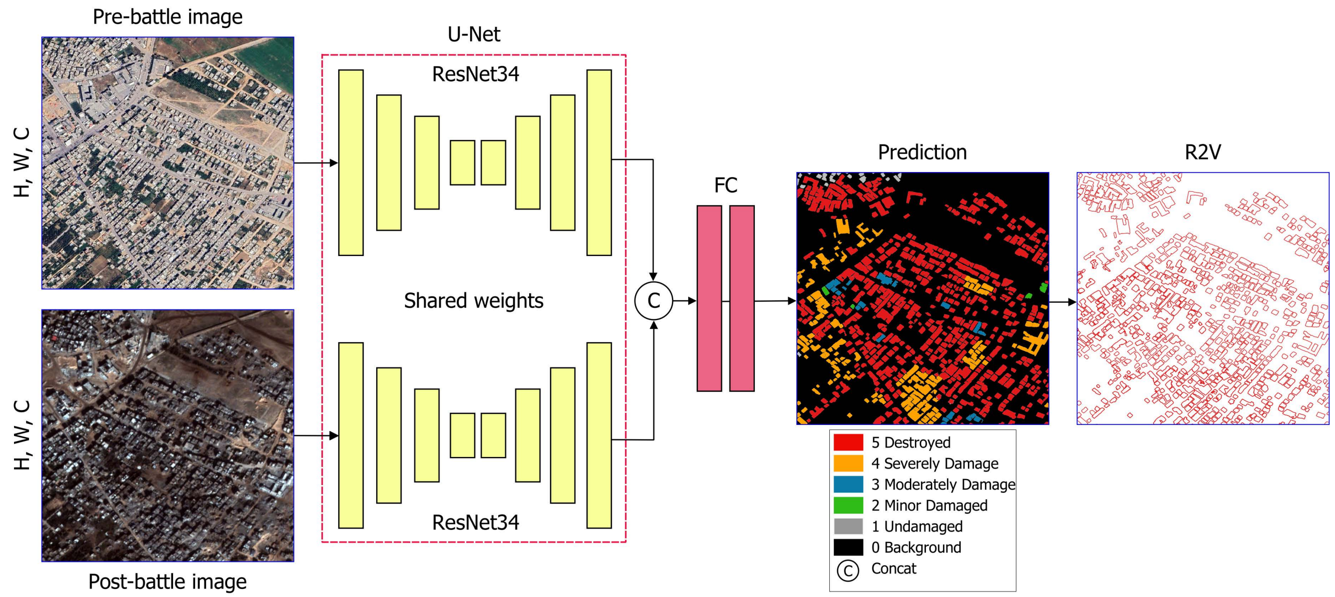

Fig. S2. Illustrates an overview of the proposed framework, featuring a U-Net model with a ResNet34 encoder. The process involves feeding the pre- and post-battle images through a Siamese network, where the output features are concatenated before reaching the final convolutional layers.

destroyed buildings using pre- and post-battle images as input. Subsequently, raster predictions are transformed into vector data (R2V). Finally, predictions of various damage states are consolidated into the vector representation of the damage estimates. Recent studies (13, 14) have indicated that the Siamese convolutional neural network is an effective method for change detection from satellite imagery. Building upon this insight, we developed a Siamese convolutional neural network with semi-supervised learning, to generate predictions for changing buildings. This network consists of two identical branches with shared weights, enabling the extraction of features from pairs of pre- and post-battle images. A U-Net-shaped segmentation model (15) with a ResNet34 (7) encoder was used as the underlying architecture for the Siamese network and pre-trained using a substantial amount of labelled disaster data. The classification of changes into five distinct damage levels was accomplished by integrating the U-Net with multi-scale feature fusion and cross-directional attention mechanisms (16). The output features from each branch are concatenated, followed by two additional fully convolutional layers. The final layer produces a building damage prediction mask. Implementation of this framework utilized an NVIDIA GRID RTX8000 (28GB) GPU. For post-predictions, the generated predictions undergo filtering through morphology with a window size of 5, and polygons with an area of less than 1 m^2 are removed. The ultimate change prediction is generated by attributing a damage attribute to each vector feature. The damage level for each vector feature is determined by the minimum majority vote of all associated pixels from the change-building prediction mask. If over 50% of the pixels within a vector feature are predicted as a specific damage level, the entire feature is assigned to that level; if there are no prediction values, its damage state is designated as an intact building. To account for the overall quality of predictions provided by the framework in detecting building damage, a manual examination of each possible building state (building destroyed/major damage/moderate/minor damage/intact building) was conducted by scrutinizing LuoJia3-01 satellite images for visibly damaged buildings. Additionally, damage scores from the UNOSAT (2) were employed for validation.

Text S5. Agricultural land changes model simulation

The conflict in Gaza has severely impacted its agricultural lands, introducing numerous detrimental dynamics, such as the destruction of irrigation infrastructure, displacement of labor due to conflict, challenges in marketing crops, bombings, bulldozer levelling, and heavy vehicle activities. Fig. S12 illustrates the extent of damage to agricultural lands caused by the movement of heavy vehicles, tanks, and extensive bulldozing. These factors contribute to negative changes in agricultural land patterns during wartime. Additionally, natural factors like rainfall and temperature variations can further degrade crop health and density. This precarious situation poses a threat to food security, leading to increased dependence on food aid and imports. In our analysis, we focused on the impact of these abnormal dynamics resulting from the ongoing conflict. Since the LuoJia3-01 images only consist of RGB bands, we employed the normalized red-green difference index (NRGDI) (17), a concept previously utilized in precision agriculture using

RGB bands. The NRGDI is calculated by Eq. 2, using the reflectance values of the red and green bands of the electromagnetic spectrum, which are captured by satellite sensors. Areas showing significant decreases in NRGDI levels indicate regions with damaged agricultural infrastructure, evidenced by lower vegetation density.

$$NRGDI = \frac{R - G}{R + G}, \quad [2]$$

$$dNRGDI = NRGDI_{pre} - NRGDI_{post}. \quad [3]$$

Where R is the reflectance in the red band, and G is the reflectance in the green band. Higher NRGDI values indicate healthier vegetation. Conversely, low NRGDI values indicate damaged vegetation.

Specifically, we created masks for agricultural land unaffected before the conflict in May 2022 by calculating the NRGDI and applying a threshold hash of 0.15 (https://analytics-library.eiwa.ag/ngrdi-normalized-green-red-difference-index/). Similarly, we created masks of farmland affected by the ongoing conflict on different dates. Next, we calculated the dNRGDI using Eq. 3 by computing the difference between two NRGDI images. This method enables the detection of changes in cultivated areas for annotation purposes without identifying specific crop types. Pixels with dNRGDI levels below a threshold value of -0.05 are classified as damaged, and those above that threshold are classified as undamaged. This threshold was determined by testing various values to find the one that best separates affected areas from unaffected areas. These labels were then used to train a machine-learning model to detect damage to cultivated agricultural lands. The analysis revealed that areas with significant changes in dNRGDI, such as the North and Gaza regions, correspond to active battlefields. Notably, the available images from the LuoJia3-01 satellite allowed for accurate visual interpretation of various land cover types. Training samples were manually collected through visual interpretation of affected and unaffected areas over two years, encompassing the pre-conflict year (2022) and the conflict years (2023-2024). Ultimately, 300,000 pixels were selected to train the model within the SNAP environment offered by the European Space Agency (ESA) (http://step.esa.int/main/).

The Random Forest (RF) classification algorithm was chosen for distinguishing between affected and unaffected farmland due to its proven effectiveness in land use land cover classification (https://github.com/samniem/Random-Forest-Land-Classification). The RF classifier underwent training on the high-performance virtual machine of Wuhan University, resulting in the creation of maps between October 2023 and February 2024. The algorithm demonstrated an accuracy of 94.7% in distinguishing between affected and unaffected agricultural lands, verified through validation samples and UNOSAT(18) data. In future work, we plan to build on our existing use of the NRGDI, which has so far only been used to create labels for conflict-affected farmland rather than to identify specific crop types. This initial step is crucial for identifying affected areas. Significant emphasis will be placed on exploring the potential productivity impacts associated with conflict on different crop species and their implications for food security and public health.

References

1. Li D, Wang M, Yang F *et al.* Internet intelligent remote sensing scientific experimental satellite LuoJia3-01. *Geo-Spat Inf Sci* 2023; **26**: 257–61.
2. UNOSAT. Gaza Strip Comprehensive Damage Assessment - January 2024. https://unosat.org/products/3793 (14 March 2024, date last accessed).
3. Saleh T, Holail S, Weng X *et al.* Ship Detection in Cosmo-Skymed SAR Imagery Using a Novel Cnn-Based Detector: a Case Study from the Suez Canal. *ISPRS Ann Photogramm Remote Sens Spatial Inf Sci* 2023; **10**: 715–22.
4. Picterra. https://github.com/Picterra (17 March 2024, date last accessed).
5. Shorten C and Khoshgoftaar TM. A survey on image data augmentation for deep learning. *J big data* 2019; **6**: 1–48.
6. Han J, Ding J, Li J *et al.* Align deep features for oriented object detection. *IEEE Trans Geosci Remote Sensing* 2021; **60**: 1–11.
7. He K, Zhang X, Ren S *et al.* Deep residual learning for image recognition. In: Proceedings of the IEEE Conference on Computer Vision and Pattern Recognition (CVPR) 2016. Las Vegas: IEEE, 770–8.
8. Lin TY, Dollár P, Girshick R *et al.* Feature pyramid networks for object detection. In: Proceedings of the IEEE Conference on Computer Vision and Pattern Recognition (CVPR) 2017. Honolulu: IEEE, 2117–25.
9. Liu S, Qi L, Qin H *et al.* Path aggregation network for instance segmentation. In: Proceedings of the IEEE Conference on Computer Vision and Pattern Recognition (CVPR) 2018. Salt Lake City: IEEE, 8759–68.

10. Hosang J, Benenson R and Schiele B. Learning non-maximum suppression. In: Proceedings of the IEEE Conference on Computer Vision and Pattern Recognition (CVPR) 2017. Honolulu: IEEE, 4507–15.
11. Tammina S. Transfer learning using vgg-16 with deep convolutional neural network for classifying images. *IJSRP* 2019; **9**: 143–50.
12. Cao J, Yan M, Jia Y *et al.* Application of a modified Inception-v3 model in the dynasty-based classification of ancient murals. *EURASIP* 2021; **2021**: 1–25.
13. Holail S, Saleh T, Xiao X *et al.* AFDE-Net: Building Change Detection Using Attention-Based Feature Differential Enhancement for Satellite Imagery. *IEEE Geosci Remote Sens Lett* 2023; **20**: 1–5.
14. Holail S, Saleh T, Xiao X *et al.* M-AFDE-NET: Novel Deep Learning-Based Building Change Detection of Freshly Built Locales from Satellite Imagery in the Nile Valley, Egypt. *Int Arch Photogramm Remote Sens Spatial Inf Sci* 2023; **48**: 1393–8.
15. Zheng X and Chen T. Segmentation of High Spatial Resolution Remote Sensing Image Based on U-Net Convolutional Networks. In: Proceedings of the IEEE International Geoscience and Remote Sensing Symposium (IGARSS) 2020. Waikoloa: IEEE, 2571–4.
16. He Y, Wang J, Liao C *et al.* MS4D-Net: Multitask-Based Semi-Supervised Semantic Segmentation Framework with Perturbed Dual Mean Teachers for Building Damage Assessment from High-Resolution Remote Sensing Imagery. *Remote Sens* 2023; **15**: 478.
17. Radočaj D, Šiljeg A, Marinović R *et al.* State of major vegetation indices in precision agriculture studies indexed in web of science: A review. *Agriculture* 2023; **13**: 707.
18. UNOSAT. Gaza Strip Agricultural Damage Assessment - January 2024. https://unosat.org/products/3792 (14 March 2024, date last accessed).

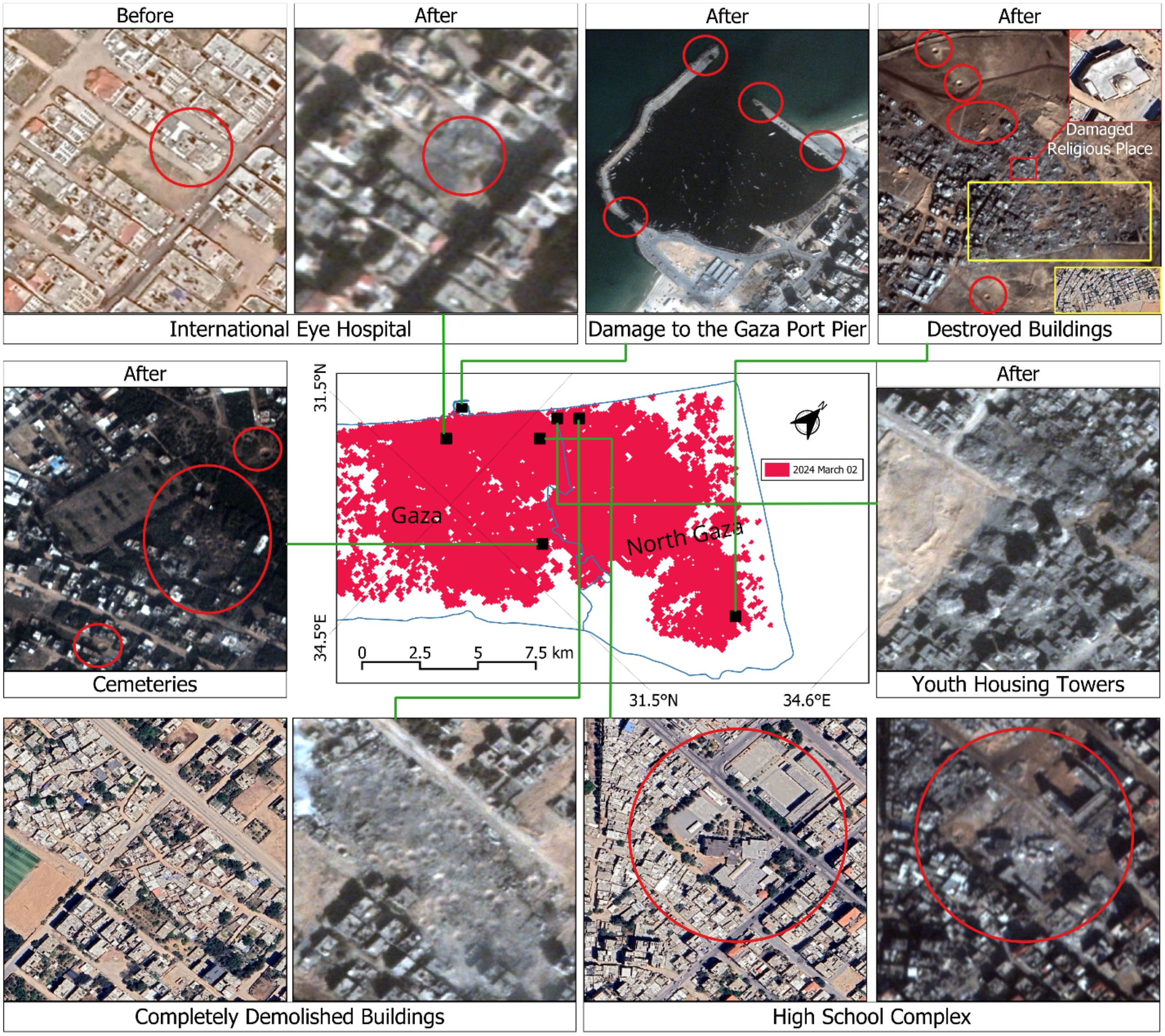

Fig. S3. Examples of vital landmarks in northern Gaza that were targeted by rocket and artillery shells, resulting in destruction. Buildings that have been damaged are depicted in dark red. The panels illustrate the contours of destroyed landmarks, including the International Eye Hospital, the Gaza Port Pier, the Omar bin Abdul Aziz Educational High School Complex, a house of worship, youth housing towers, and a cemetery that sustained damage. Sources: Google Earth/LuoJia 3-01 Satellite Technologies. Author creations using QGIS 3.32.

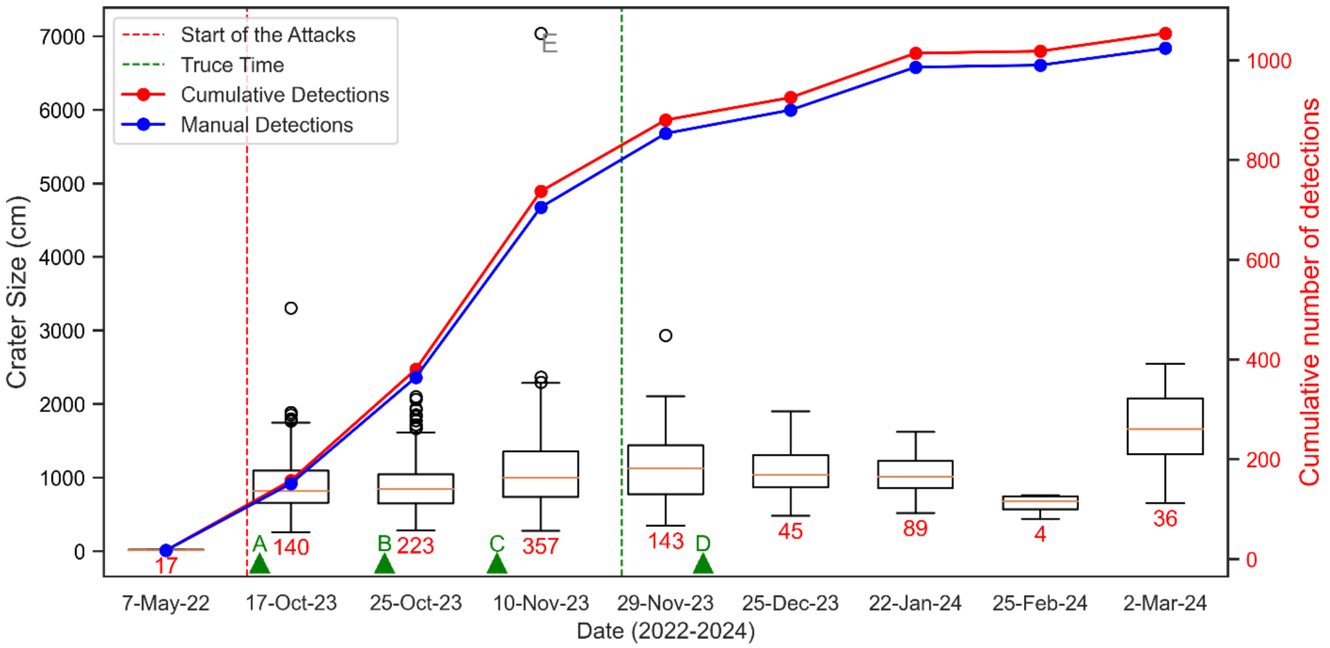

Fig. S4. Timeline for detecting missile craters in the northern Gaza area. This chart delineates the number of crater detections alongside their respective sizes. The cumulative number of detections is represented by a red line, whereas the box plots illustrate the varying sizes of craters, indicated by the black values. The blue values beneath the boxes denote the number of detections for each time period. The dotted red line marks the onset of the attacks (October 7, 2023), and the dashed green line indicates the declaration of a truce (spanning 4 days) to facilitate prisoner exchanges between both parties. Green arrows highlight specific instances of craters and breaches in the Israeli wall, as depicted in Fig. S5. Sources: Author calculations.

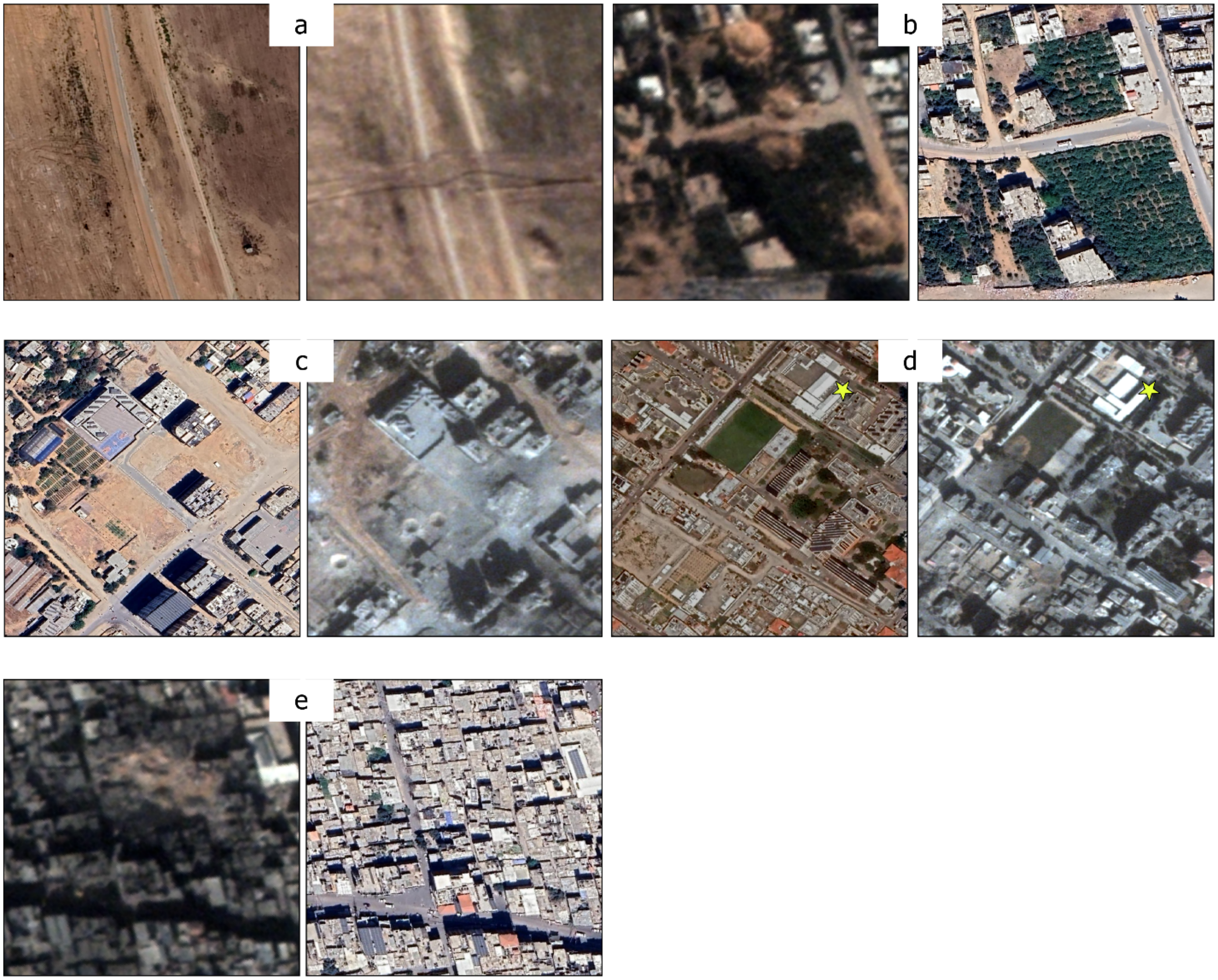

Fig. S5. Illustrates examples of missile craters and their impacts on infrastructure. a, illustrates the crossing of an iron fence by Hamas on October 7, 2023. b, depicts significant craters affecting agricultural lands. c, details the complete destruction of the building belonging to the Palestinian Charitable Care Society. d, Damages of Al-Azhar Islamic University. e, describes a severe bomb detonation at Jabalia refugee camp, resulting in a crater comparable in size to a large muddy water pool. Sources: Author creations using QGIS 3.32 based on Google Earth/LuoJia 3-01 Satellite Technologies.

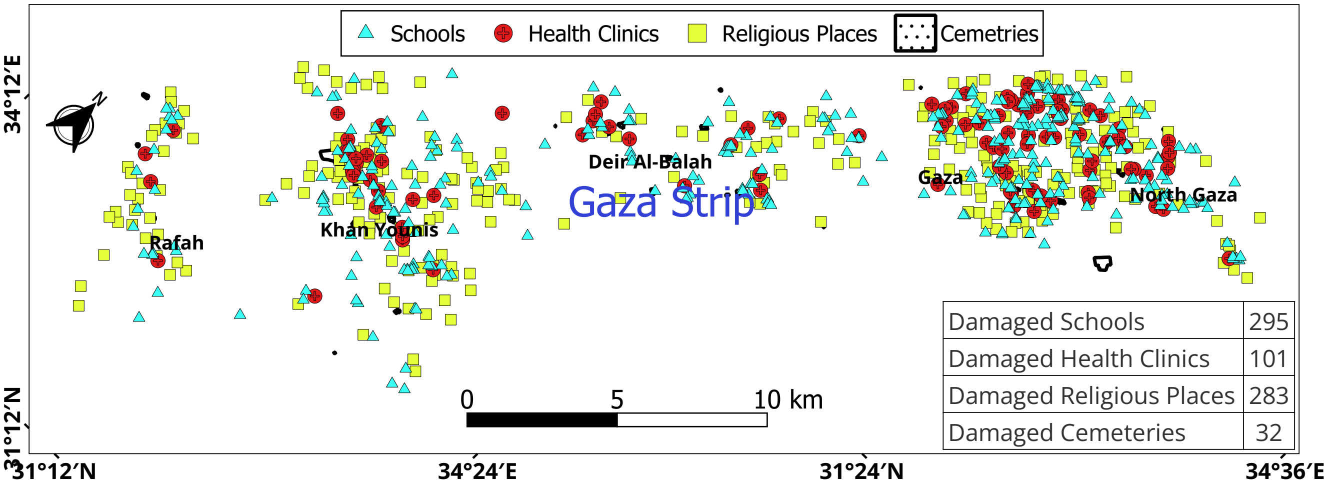

Fig. S6. A map of the numbers and distribution of different types of buildings damaged by the war for March 02, 2024. Among them are hospitals and medical clinics, places of worship such as mosques and churches, educational institutions including kindergartens, schools and universities, and cemeteries for burying the dead. These numbers represent a direct result of the brutal actions taken by the Israeli occupation army against the Palestinian people. Source: Author creations using QGIS 3.32.

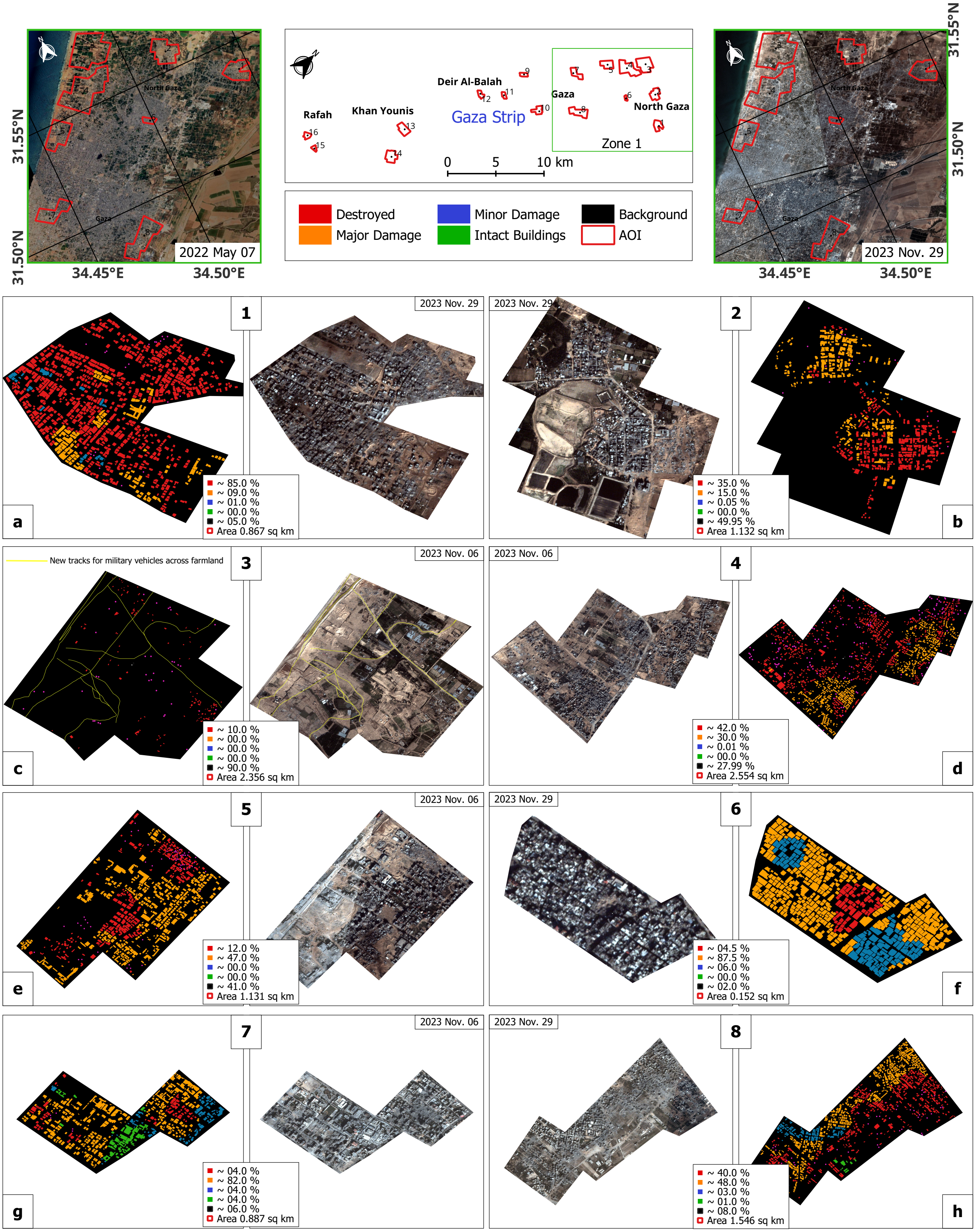

Fig. S7. Presents the detection map with multi-level damages in zone 1 of the Gaza Strip. The detection is concentrated on distinct areas, as indicated at the top of the map with zone one, and highlighted with green square for zone one which encompasses North Gaza and Gaza City (1-8), eight area of interest are presented for visualizing the detection there. Different levels of damage are visually represented using colors, designating destroyed areas in red, intact buildings in green, minor damaged buildings in blue, and major damage in orange.

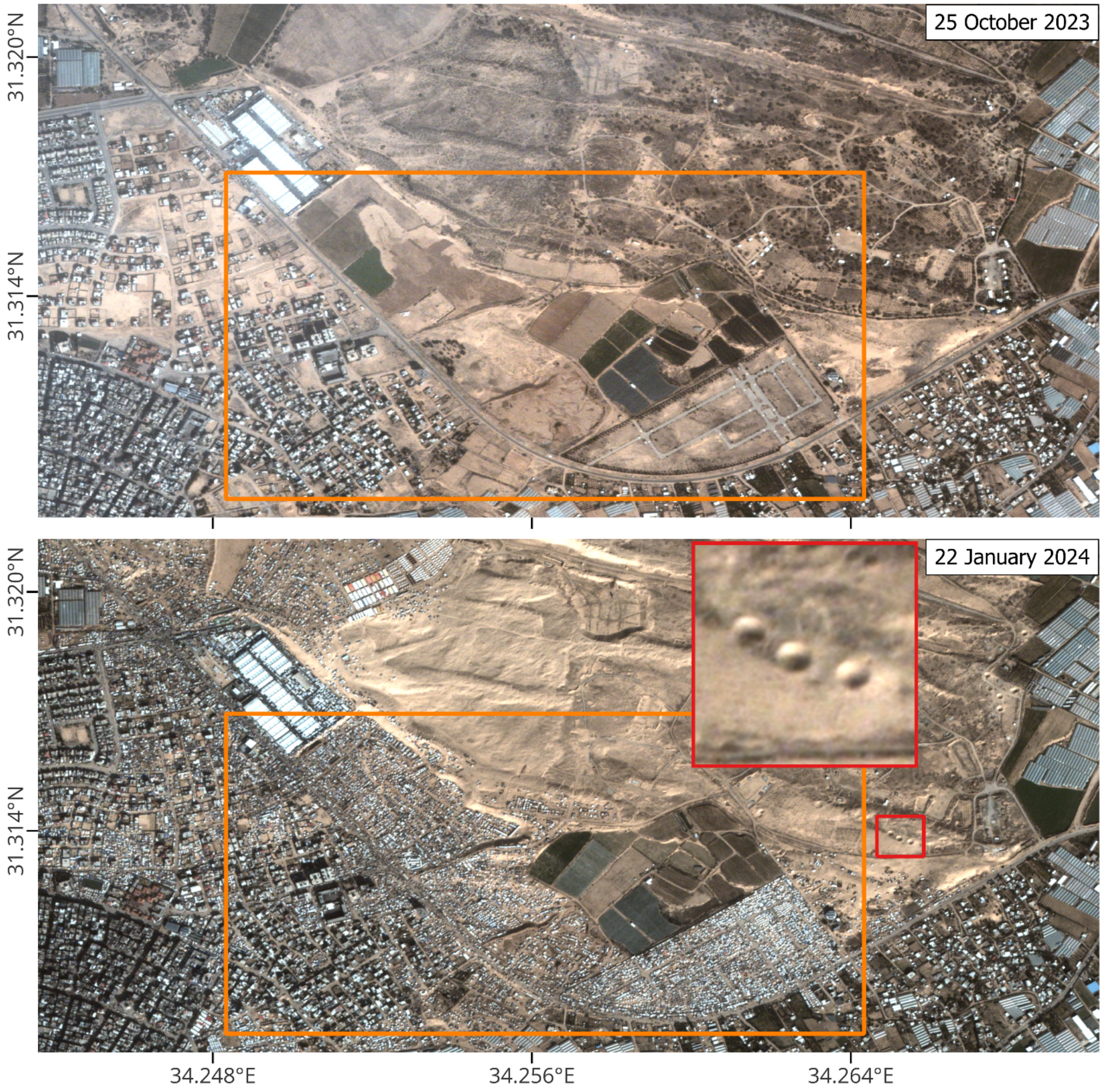

Fig. S8. Presents LuoJia3-01 satellite imagery illustrating the congestion in a section of Rafah city in the southern Gaza Strip, densely populated with tents sheltering displaced individuals. The initial image was captured 18 days following the conflict's onset, whereas the subsequent image was taken approximately four months later. An orange rectangle highlights an area adjacent to agricultural fields that initially appears vacant but is later depicted as a densely populated tent city in the more recent image. Surrounding the temporary shelters for displaced persons are numerous missile craters, with the red rectangle indicating areas where civilian casualties may have occurred. Sources: Author creations using QGIS 3.32 based on LuoJia3-01 satellite imagery.

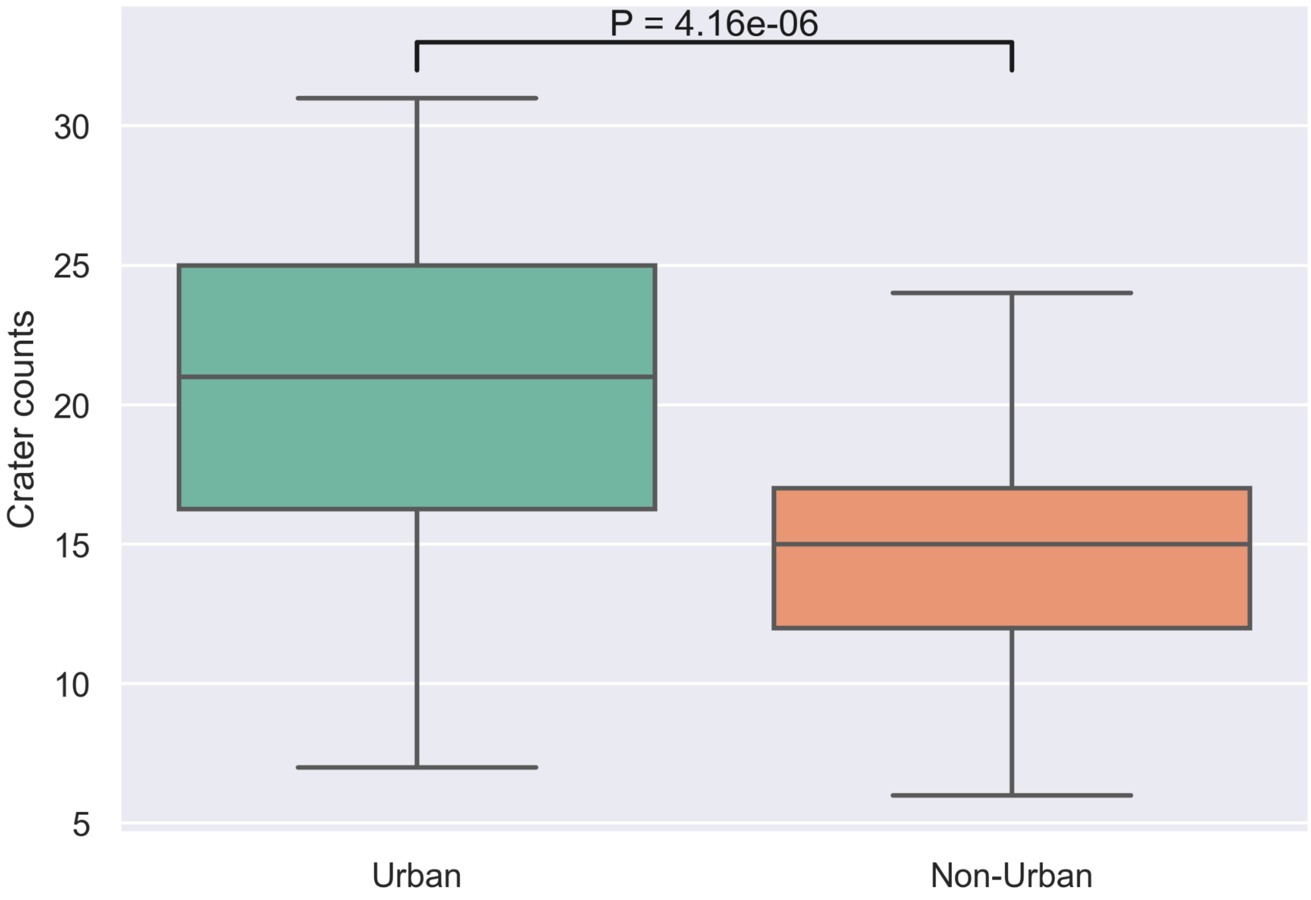

Fig. S9. Comparison of the numbers of missile craters detected in urban and non-urban areas. Eighty-four missile crater samples were selected, and the boundaries of each sample were manually delineated based on LuoJia3-01 satellite imagery. Boxplots represent the average number of missile craters during different periods in urban (n = 605 pixels) and non-urban areas (n = 8,352 pixels). Comparative analysis was performed using Welch's t-test ($P < 0.001$) at a 95% confidence level, indicating a significant difference in the number of craters between the two samples. The bar in the middle of the boxplot represents the median value, while the upper and lower bounds represent the first and third quartiles, respectively; the whiskers indicate the minimum and maximum values. The boxplots demonstrate that densely populated areas exhibit a higher mean and greater variance in the number of missile craters compared to less populated areas. Sources: Author calculations.

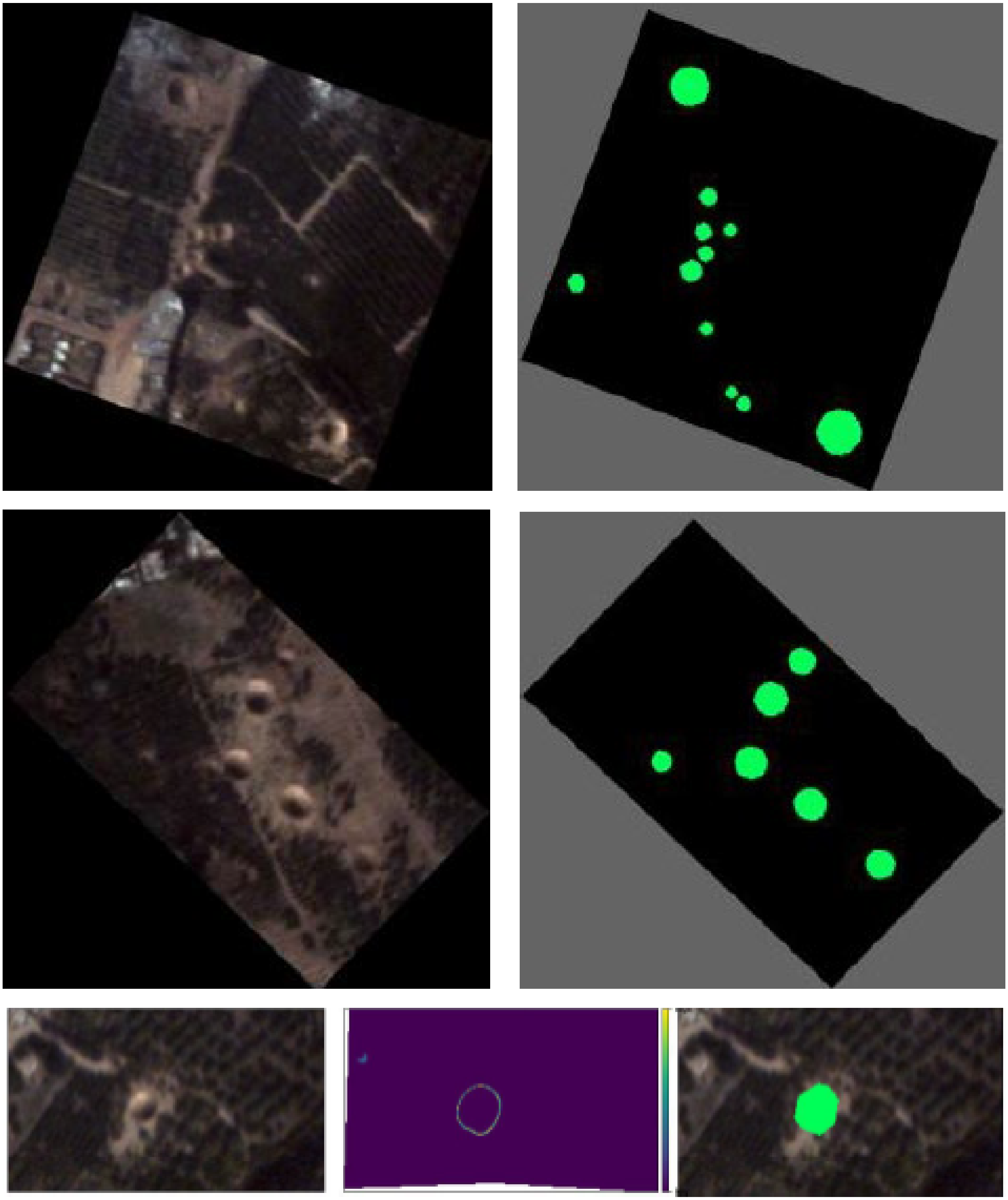

Fig. S10. Examples of missile craters and their corresponding masks in the training dataset. The first and second rows display various sizes of precisely annotated craters. The third-row presents, from left to right, the original RGB image, the uncertainty map, and the detected craters. Category labels are as follows: black for the background and grey for padding.

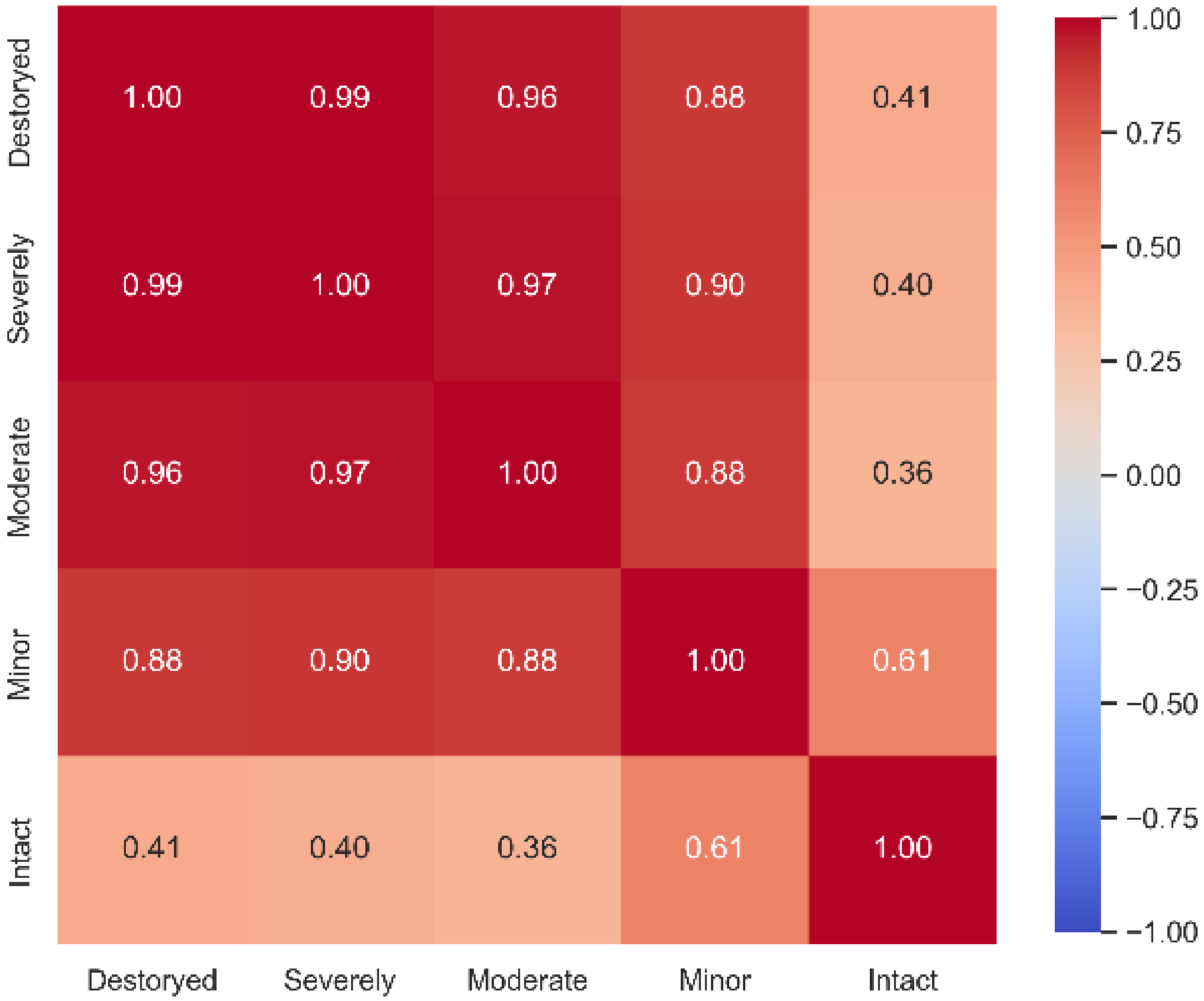

Fig. S11. Correlation matrix between different damage levels. Colors range from dark red (high positive correlation) to light red/pink (lower positive correlation). There are strong correlations between the first three damage categories, indicating a consistent assessment across these levels, while intact structures show a significantly lower correlation with the damaged categories, indicating a clear distinction in the dataset between damaged and undamaged buildings.

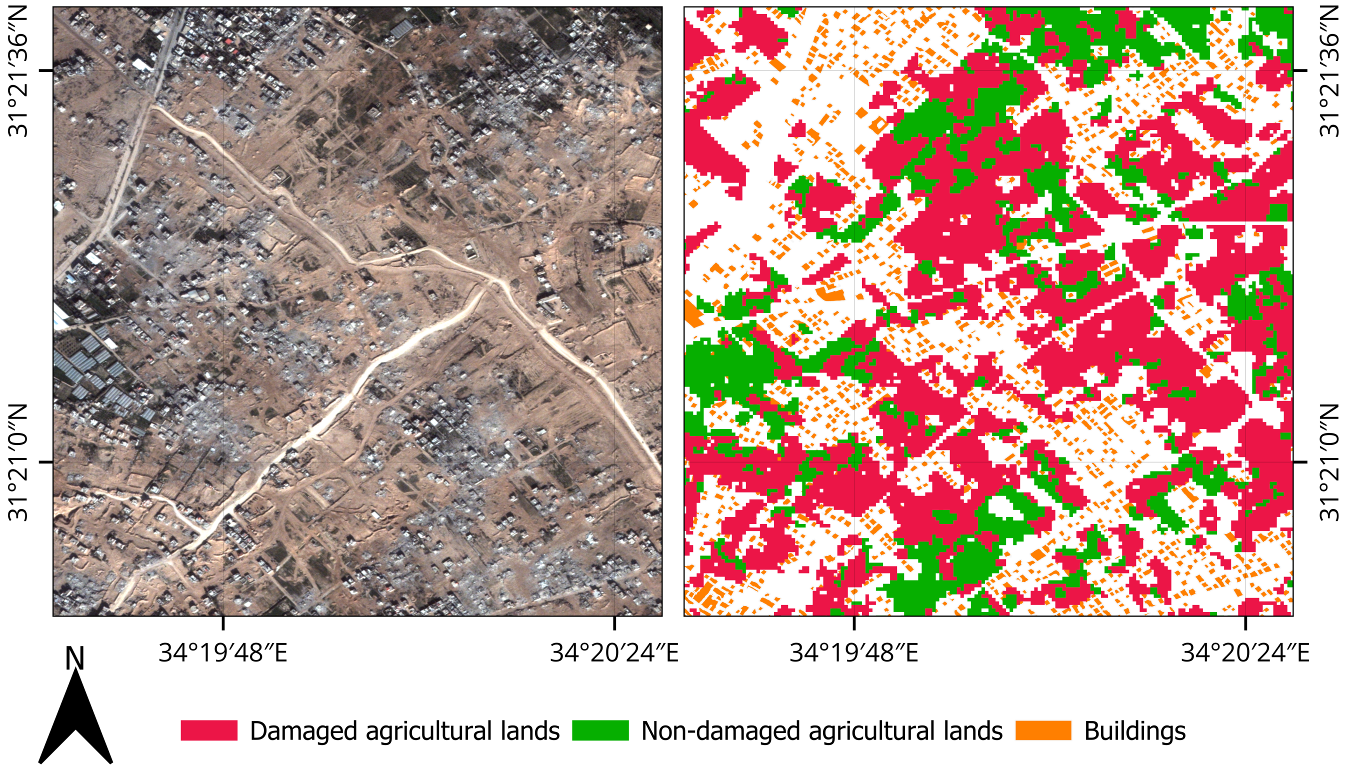

Fig. S12. Shows the extent of damage to agricultural land in a rural area in Khan Yunis Governorate as a result of severe bulldozing and the creation of paths for the movement of heavy machinery and tanks in preparation for the establishment of a buffer zone separating northern Gaza from its center. The damage map was created using the proposed machine learning approach, and the maps were output within the QGIS software package.

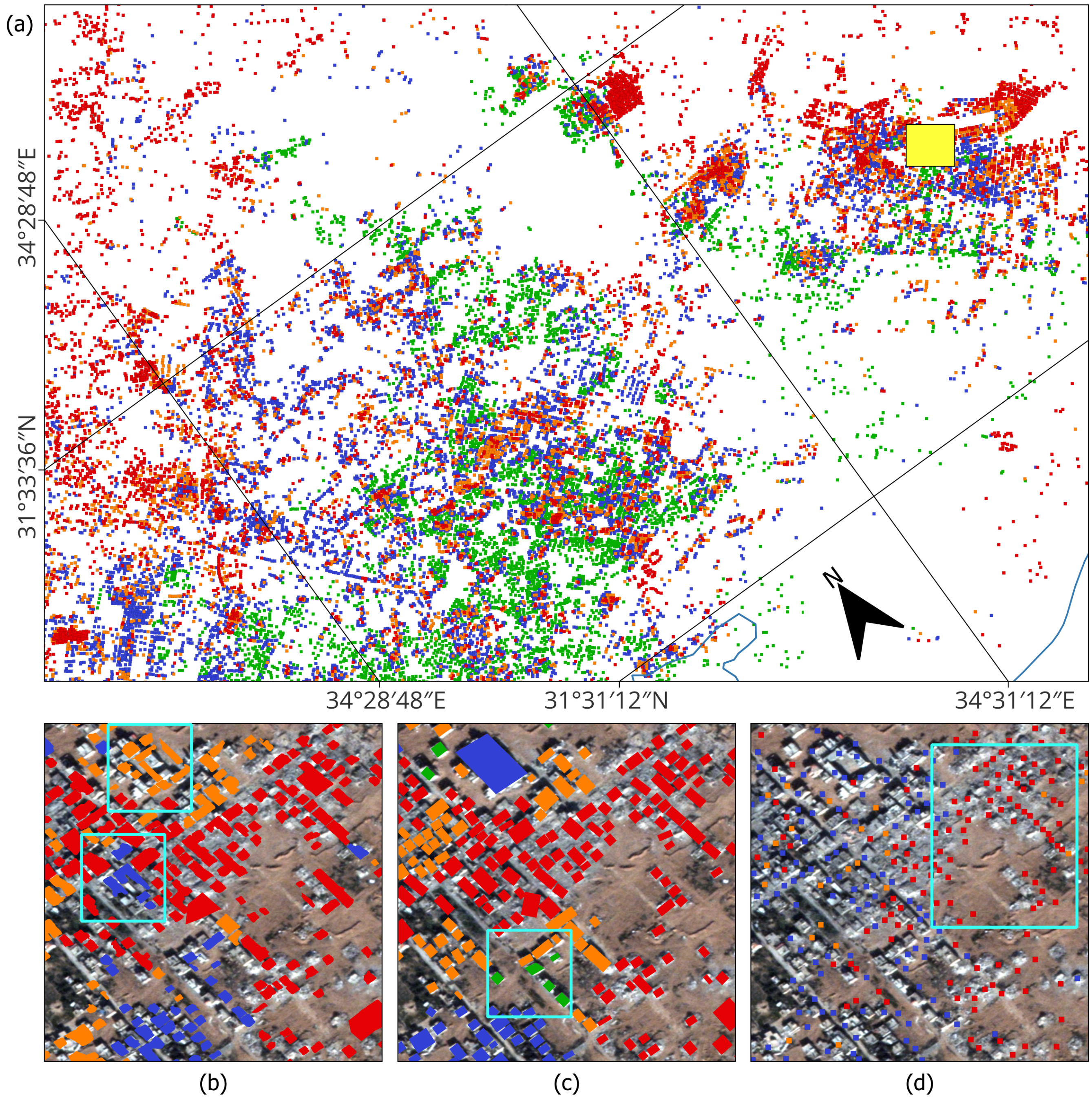

Fig. S13. The labels of the North Gaza Governorate on November 29, 2023. Red indicates destruction, orange indicates major damage, blue indicates minor damage, and green indicates no damage. The yellow rectangle includes a populated residential area in Beit Hanoun under analysis, which was heavily damaged, as shown in the zoomed-in images in (b–d). Panels (b) and (c) show the ground truth damage annotations before and after manual examination, respectively. Panel (d) shows the UNOSAT damage annotations, providing an additional layer of verification. The automated model may classify a building as slightly damaged, but after manual examination, it may be identified as undamaged by noting the entire structure that the model analysis neglects, as shown by the green color in panel (c). We can observe that the damage annotations points indicated in red by UNOSAT consistent with the pre- and post-manual examination model with slight differences, as shown in the cyan rectangle in panel (d). Source: Mapping conducted by the author using QGIS 3.32 and LuoJia3-01 satellite imagery.

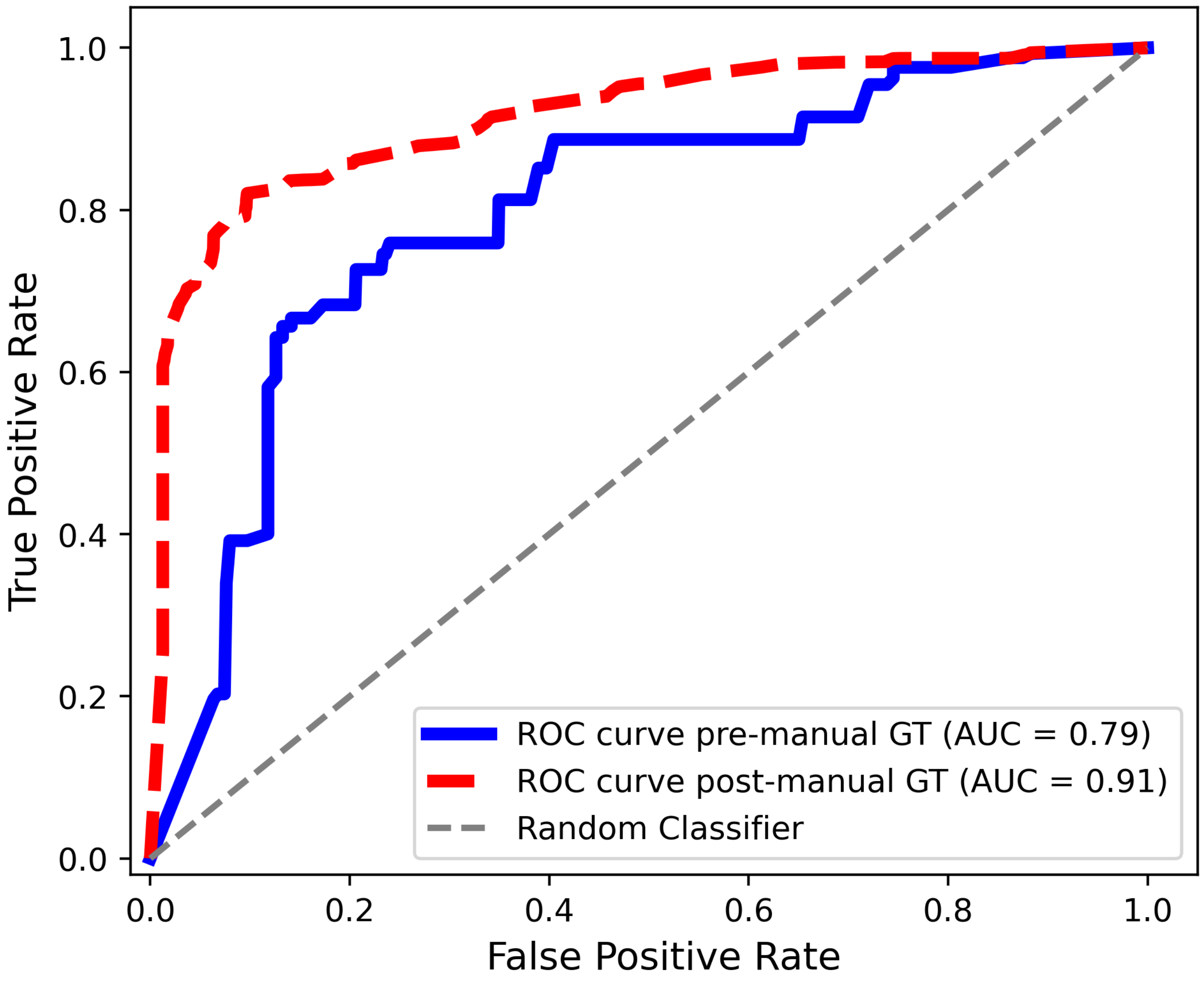

Fig. S14. ROC curves before and after manual examination in portions of the conflict area not included in the training sample, specifically within the Khan Yunis Governorate in Gaza. The performance depicted represents 25% of the test sample used for validation. The proposed model, following manual examination, achieves a high accuracy score with an AUC of 0.91, significantly improving upon the pre-manual examination AUC of 0.79. This enhancement yields practically feasible destruction data. Sources: Author calculations.

Table S1. Specifications of high-resolution satellite imagery utilized in the study.

Attribute	LuoJia3-01 (LJ03-1)
Affiliation	Wuhan University, China
Launch Date	January 15, 2023
Observation Period	October 17, 2023 - March 2, 2024
Resolution	0.65 meters
Sensor	OIT
Product Level	L2A
Format	GEOTIFF
Imagery Type	Optical
Average Image Size	480 MB
Pixel Count per Image	315 million
Color Bands 3	(RGB)
Targeted Area	Gaza Strip
Total Images Captured	40
Data Processing UTM Zone	36 N

Table S2. Tabulated sample of missile craters detected in the Gaza Strip: classification by region, size, and date as presented in Fig. S3.

Region	Size (m)	Latitude (N)	Longitude (E)	Date	Category	F1-score
North Gaza	33.0	31.537853	34.531016	17 October 2023	Large	95.3 %
Rafah	15.2	31.282107	34.280863	25 October 2023	Medium	89.4 %
North Gaza	70.4	31.533284	34.498638	10 November 2023	Very Large	96.3 %
North Gaza	18.6	31.559351	34.479681	25 December 2023	Medium	90.6%
Gaza City	19.7	31.482573	34.458199	1 January 2024	Medium	88.1%
Khan Younis	16.4	31.337434	34.317259	21 January 2024	Medium	91.5 %
North Gaza	6.1	31.500202	34.508653	25 February 2024	Small	73.8 %
Deir-Al-Balah	13.5	31.396415	34.364068	25 February 2024	Medium	85.7 %
North Gaza	17.9	31.526776	34.490266	2 March 2024	Medium	89.2 %

Table S3. Tabulated number of missile craters detected in five governorates of the Gaza Strip.

Region	Area (km2)	Crater
North Gaza	61.14	1036
Gaza	73.86	1238
Deir Al-Balah	58.39	384
Khan Younis	107.62	872
Rafah	64	217
**Gaza Strip**	**365**	**3747**

Table S4. Model results before and after manual examination of ground truth for buildings with varying levels of damage in North Gaza.

Damage Level	(1) Before Manual GT	(2) After Manual GT	(3) Before Manual GT %	(4) After Manual GT %	(5) Mean Absolute Error %	(6) Squared Error %
Destroyed	8100	7421	22.69	20.78	1.91	3.65
Severely damaged	12960	11270	36.30	31.57	4.73	22.37
Moderately damaged	9740	10031	27.28	28.10	0.82	0.67
Minor damage	4900	6978	13.73	19.55	5.82	33.87
Total damage	35700	35700	100	100	13.28	60.56
Mean Squared Error (MSE)			15.14 %			
Root Mean Squared Error (RMSE)			**3.89 %**			
R-squared			**76.82 %**			
Overall Accuracy			**93.34 %**			

Sources: Author calculations based on LuoJia3-01 satellite imagery and model damage annotations before and after manual examination. Note: Columns (1) and (2) present the number of ground truth (GT) labels for various damage levels generated by the model before and after manual examination. Columns (3) and (4) display the percentages of each damage type in these two cases. Column (5) indicates the difference between the GT values before and after manual examination, measured using mean absolute error (MAE). Column (6) shows the squared error used in the calculation of the root mean square error (RMSE). The MAE percentages between the pre- and post-examination values differed by approximately 3.89%, which can be considered reasonably accurate given the high overall accuracy of about 93.34%. The R-squared value of 76.82% suggests a strong fit, indicating that the damage levels before examination are closely related to those after examination. Consequently, the model demonstrates an acceptable degree of confidence in predicting future damage levels.

Table S5. Validation accuracy result of craters detection model with different backbones (30 epochs) using the Luojia3-01 satellite imagery of North Gaza, Palestine.

Backbone	November 29, 2023 imagery			January 1, 2024 imagery		
	P	R	F1	P	R	F1
ResNet34	93.229 %	85.406 %	89.146 %	94.421 %	87.065 %	90.594 %
VGG16	89.785 %	80.514 %	84.897 %	88.657 %	81.474 %	84.914 %
InceptionV3	87.174 %	78.795 %	82.773 %	87.538 %	79.105 %	83.108 %

Movie S1. Video Demo
